# *Schizochytrium* sp. (T18) Oil as a Fish Oil Replacement in Diets for Juvenile Rainbow Trout (*Oncorhynchus mykiss*): Effects on Growth Performance, Tissue Fatty Acid Content, and Lipid-Related Transcript Expression

**DOI:** 10.3390/ani11041185

**Published:** 2021-04-20

**Authors:** Angelisa T. Y. Osmond, Michael T. Arts, Jennifer R. Hall, Matthew L. Rise, Richard P. Bazinet, Roberto E. Armenta, Stefanie M. Colombo

**Affiliations:** 1 Department of Animal Science and Aquaculture, Dalhousie University, Truro, NS B2N 5E3, Canada; angelisa.osmond@dal.ca; 2 Department of Chemistry and Biology, Ryerson University, Toronto, ON M5B 2K3, Canada; michael.arts@ryerson.ca; 3 Aquatic Research Cluster, CREAIT Network, Ocean Sciences Centre, Memorial University of Newfoundland, St. John’s, NL A1C 5S7, Canada; jrhall@mun.ca; 4 Department of Ocean Sciences, Memorial University of Newfoundland, St. John’s, NL A1C 5S7, Canada; mrise@mun.ca; 5 Department of Nutritional Sciences, University of Toronto, Toronto, ON M5S 1A8, Canada; richard.bazinet@utoronto.ca; 6 Mara Renewables Corporation, Dartmouth, NS B2Y 4T6, Canada; rarmenta@maracorp.ca; 7 Department of Process Engineering and Applied Science, Dalhousie University, Halifax, NS B3H 4R2, Canada

**Keywords:** EPA, DHA, microbial oil, salmonid

## Abstract

**Simple Summary:**

One of the main concerns in aquaculture is the overreliance on wild-sourced fish oil as the main source of omega-3 fatty acids in diets for farmed fish. Microbes, such as *Schizochytrium*, naturally produce high levels of omega-3 fatty acids that could potentially replace fish oil in aquaculture feeds. In this study, we tested the oil from a new strain of *Schizochytrium* (T18) to replace fish oil in diets for farmed rainbow trout and looked at fish growth performance, muscle and liver fatty acid content, and the expression of transcripts involved in fat metabolism. Trout were raised for 8 weeks and fed diets with either: (1) fish oil control, (2) low inclusion of microbial oil, or (3) higher inclusion of microbial oil. Inclusion of *Schizochytrium* sp. (T18) oil at high or low levels in the diet resulted in a similar growth performance as seen in trout fed the control diet; however, muscle and liver fatty acid profiles were impacted by diet. Overall, our results showed that *Schizochytrium* (T18) is an effective source of omega-3 fatty acids in diets for rainbow trout.

**Abstract:**

In this study, we evaluated whether oil extracted from the marine microbe, *Schizochytrium* sp. (strain T18), with high levels of docosahexaenoic acid (DHA), could replace fish oil (FO) in diets for rainbow trout (*Oncorhynchus mykiss*). Three experimental diets were tested: (1) a control diet with fish oil (FO diet), (2) a microbial oil (MO) diet with a blend of camelina oil (CO) referred to as MO/CO diet, and (3) a MO diet (at a higher inclusion level). Rainbow trout (18.8 ± 2.9 g fish^−1^ initial weight ± SD) were fed for 8 weeks and evaluated for growth performance, fatty acid content and transcript expression of lipid-related genes in liver and muscle. There were no differences in growth performance measurements among treatments. In liver and muscle, eicosapentaenoic acid (EPA) was highest in trout fed the FO diet compared to the MO/CO and MO diets. Liver DHA was highest in trout fed the MO/CO diet compared to the FO and MO diets. Muscle DHA was highest in trout fed the MO and MO/CO diets compared to the FO diet. In trout fed the MO/CO diet, compared to the MO diet, *fadsd6b* was higher in both liver and muscle. In trout fed the FO or MO/CO diets, compared to the MO diet, *cox1a* was higher in both liver and muscle, *cpt1b1a* was higher in liver and *cpt1a1a*, *cpt1a1b* and *cpt1a2a* were higher in muscle. *Schizochytrium* sp. (T18) oil was an effective source of DHA for rainbow trout.

## 1. Introduction

One of the central concerns in aquaculture is the overreliance on wild-sourced fish oil (FO) as a primary source of dietary omega-3 (n-3) long-chain polyunsaturated fatty acids (LC-PUFA) [1]. Unlike FO, which is a primary source of marine-derived LC-PUFA, namely eicosapentaenoic acid (EPA; 20:5n-3) and docosahexaenoic acid (DHA; 22:6n-3), the inclusion of alternative terrestrially sourced oils in formulated aquaculture diets typically do not contain these essential n-3 LC-PUFA [2]. Both EPA and DHA contribute substantially to optimal fish growth, development, inflammatory response, and neural and ocular tissue function [3]. Inclusion of n-3 LC-PUFA in salmonid diets is recommended at a rate of 1–2%, depending on species and stage of development [4]. For humans, consumption of fish and fish-based products remains the primary source of essential n-3 LC-PUFA necessary for optimum health [5]. The n-3 LC-PUFA contributes substantially to human growth and development, affecting immune system function, cardiovascular and neurological health, retinal health, membrane fluidity, cellular metabolism, and inflammation [5,6]. Dietary DHA, in particular, has also been closely linked to healthy fetal development during pregnancy, infant behavior, mood, and cognitive function [7,8,9]

In aquatic ecosystems, marine microbes play an important role in producing several high-value nutritional compounds such as EPA and DHA [5,10,11,12]. The active biosynthesis of compounds such as n-3 LC-PUFA by microorganisms is an endogenous response to growth, development, and changing environmental conditions such as temperature [13]. Fish typically rely on dietary n-3 LC-PUFA, such as EPA and DHA, due to their limited capacity to biosynthesize these compounds [10,11]. As such, fish obtain these nutrients as they are transferred from primary producers (and other microbes) along the food chain [10,11]. Derived microbial oils (MO) from microorganisms have become of great interest as sustainable and advantageous alternatives to FO use in aquaculture and other industries [14].

Recent research has focused on the incorporation of microbially derived n-3 LC-PUFA for aquafeeds and other industrial applications due to their capacity to be produced through efficient and sustainable means [13,15,16,17]. In this application, microbially sourced n-3 LC-PUFA provide an opportunity to bypass food chain nutrient transfers and instead provide fish with a dietary source of n-3 LC-PUFA from microbial species reared within a closed system [18]. This has the added benefit of reducing exposure to environmental contaminants, such as heavy metals, PCBs, pharmaceuticals, etc., that can be present in fish-derived feed products [19,20]. Many microbes produce high amounts of total lipids, contributing to 30–70% of their total dry weight (10–50% wet weight) content [13]. In the past 5 years, various strains of *Schizochytrium* spp. have been evaluated for their applications in aquafeed production [14,21]. Members of the genus *Schizochytrium* are unicellular eukaryotic organisms that belong to the family *Thraustochytriacea* in the kingdom *Stramenopila* [21]. Often mistaken for microalgae, these non-photosynthetic organisms lack cellular plastids and are taxonomically identified as marine or brackish water fungal-like protists [14,21].

A novel strain of *Schizochytrium* sp. (T18) has gained attention as a potential alternative and sustainable source of LC-PUFA [14,20,22]. When provided with adequate nutrient supplementation from nitrogen and carbon sources and under ideal growth conditions, thraustochytrids such as *Schizochytrium* spp. produce high concentrations of lipid through the use of both the Type I fatty acid synthase or the polyketide synthase pathway [15,23,24]. Biosynthetic production of lipids in thraustochytrids has been observed at >70% of their cellular mass (dry weight), and their overall lipid profile is naturally high in n-3 LC-PUFA [14]. *Schizochytrium* spp. can accumulate DHA at 30–40% of total fatty acids, making them a potential candidate for industrial production of DHA as a substitute for DHA in FO [13,25]. Due to its high DHA content, there is commercial interest in food-grade DHA products, as well as feedstock for large aquafeed producers [26].

Previous research has shown that the whole-cell biomass and extracted oil of *Schizochytrium* sp. is highly digestible by juvenile Atlantic salmon (*Salmo salar*) [27,28], including the T18 strain used in the present study [14]. In addition, DHA content was significantly higher in muscle of salmon fed diets containing 100% replacement of FO with *Schizochytrium* sp. oil [28].

Recent research has evaluated the application of the T18 strain oil in diets for Atlantic salmon parr [20], where the replacement of FO with T18 strain oil resulted in no differences in weight gain, growth rate, condition factor or feed conversion ratio and FA content was also noted to reflect dietary composition [20]. In rainbow trout, dietary inclusion of *Schizochytrium* sp. oil (Veramaris^®^, not T18 strain) showed high digestibility, the FA profiles of trout fillets were reflective of experimental diets, and no adverse effects were observed in trout growth, blood parameters, or feed conversion ratio relative to the control treatment [29]. Although this was the first study to evaluate *Schizochytrium* sp. in diets for rainbow trout, the strain evaluated in Santigosa et al. (2020) [29] was quite different than the one we used here in that it contained relatively high levels of EPA. However, *Schizochytrium* sp. T18 oil, which is high in DHA (43.4% total fatty acids) and low in EPA (0.7% total FA) has yet to be evaluated in diets for rainbow trout.

Rainbow trout are one of the most widely farmed aquaculture species in the world. Globally, production of farmed rainbow trout has risen from 752,000 tonnes in 2010 to 848,000 tonnes in 2018 and now contributes 1.6% to the total tonnage of aquaculture species worldwide, and some of the major producers include Chile, Norway, France, Italy, Spain, Denmark, USA, Canada, Germany, Iran, and the UK [30]. In Canada, production exceeded 9000 tonnes, and revenue from fish sales totalled 56.8 million CAD in 2018 [31]. In the USA, >21,000 tonnes were produced with revenue from fish sales totalling 95.6 million USD in 2019 [30,32]. Rainbow trout require dietary supplementation of n-3 LC-PUFA of up to 1% of the dry diet [4]. Although rainbow trout have the ability to synthesize n-3 LC-PUFA when provided with the n-3 precursor, alpha-linolenic acid (ALA; 18:3n-3), their capacity to do so is limited [33]. Therefore, n-3 LC-PUFA must be supplied in the diet for intensive aquaculture purposes.

In the current study, we evaluated a novel strain of *Schizochytrium* sp. (T18) as a lipid source (high in DHA, little EPA) to completely replace FO in diets for juvenile rainbow trout by assessing growth performance, muscle and liver FA content and transcript expression of lipid-metabolism-related genes. With the application of novel microbial oils solely containing high DHA and little EPA, it is essential to understand how it is metabolized and used compared to FO (the industry standard). As such, the overall aim of this study was to evaluate the impact of this novel high DHA MO on the growth, metabolism, and fatty acid content (nutritional quality) of farmed juvenile rainbow trout.

## 2. Materials and Methods

### 2.1. Microbial Oil

The extracted microbial oil (MO) used in this study was produced heterotrophically from a thraustochytrid strain identified as *Schizochytrium* sp. by Mara Renewables (Dartmouth, NS, Canada). The variety used in this study was DHA-02122016-C3 [14,20]. The FA content of the oil contained 0.7% EPA and 43.4% DHA, for a total n-3 LC-PUFA proportion of 44.1% (presented as % and µg/mg in Table 1). The MO also included low levels of ALA (0.1%), linoleic acid (LNA; 18:2n-6; 0.3%) and oleic acid (18:1n-9: 0.6%). While we were unable to quantify 22:5n-6 in this analysis, we have previously reported that the T18 strain (produced by the same company, Mara Renewables) contains 7.6% 22:5n-6 [20]. The sum of saturated FA (SFA), monounsaturated FA (MUFA) and PUFA were identified in the following proportions: 46.7%, 8.2% and 45.2%, respectively. Total n-3 and total n-6 were 44.5 % and 0.7%, respectively. The n-3/n-6 ratio for the oil was 4.5.

### 2.2. Experimental Diet Formulation and Composition

All diets were formulated to be isonitrogenous and isolipidic to meet the nutritional requirements of rainbow trout [4]. Diets were produced at the Chute Animal Nutrition lab, Faculty of Agriculture, Dalhousie University (Truro, NS, Canada). Three experimental treatments were produced as follows (Table 2), with the total added oils as 150 g/kg (Table 2): a control diet with fish (herring) oil (FO diet; 100 g/kg FO and 50 g/kg camelina oil; CO), a lower level MO and camelina oil (CO) blend (MO/CO; no FO, with 75 g/kg each of MO and CO) and a higher MO inclusion diet (MO; no fish oil, with 100 g/kg MO and 50 g/kg CO). The MO/CO diet was designed to replace FO with MO but did not fully replace other lipid sources (i.e., CO), as an example of a potential modern commercial feed; while the MO diet fully replaced FO with MO and was a major proportion of the total lipid in the diet. Diet mash was extruded using a laboratory extruder (AMANDUS KAHL GmbH and Co. KG, Dieselstraße 5–9, D- 21465, Reinbek, Germany) with a 2.0 mm die. Pellets were dried in a JWP ST series industrial cabinet oven at 65 °C for 4–5 h. Excessive fines were sifted using both a 2.5 mm and 3 mm sieve. Final pellet size was 2.8 mm. Diets were vacuumed coated at a pressure of −0.9 bar at 70 °C with added experimental oil blends at 150 g/kg of the diet. Pellets were held for 5 min at −0.9 bar, then slowly released over a 5-min period until pellets reach atmospheric pressure to ensure optimum absorption of fats into pellets. Diets were stored at −20 °C in airtight containers to reduce oxidization of fats until needed. Diets were only exposed to room temperature during periods of feeding. The final extruded diets were analyzed for nutritional composition at the Nova Scotia Department of Agriculture Laboratory Services (Truro, NS, Canada; Table 3).

### 2.3. Experimental Fish

Juvenile rainbow trout (*n* = 225; 18.8 ± 2.9 g fish^−1^ initial weight ± SD) were obtained from North River Fish Farms (North River, NS, Canada) and transported to the Aquaculture lab, Faculty of Agriculture, Dalhousie University (Truro, NS, Canada). Guidelines for the ethical treatment of fish were followed, in accordance with the Canadian Council of Animal Care (Dalhousie University-approved protocol 2019-101). The rainbow trout were randomly distributed into 9 circular fiberglass experimental tanks (232 L per tank), with each tank containing 25 fish. Each experimental diet was fed to triplicate tanks, with tank as the experimental unit (*n* = 3). A flow-through supply of freshwater from a local aquifer was supplied to the tank system at a rate of 2–3 L min^−1^. The dissolved oxygen (10 mg L^−1^; 100%) and approximate water temperature (14 ℃) were monitored and recorded daily. Fish were reared on a natural photoperiod over the 8-week trial (average 12 h light: 12 h dark). Fish were fed twice daily to apparent satiation and feed intake was recorded weekly. Any observed mortalities were weighed and recorded throughout the experimental trial.

### 2.4. Tissue Sampling

Sampling of fish occurred initially at week 0, the day before feeding the experimental diets and at week 8. At week 0 and week 8, four fish per tank were randomly netted and euthanized with an overdose of anesthetic (tricaine methane sulfonate; MS-222, Syndel, Nanaimo, BC, Canada) and clinical signs of death were confirmed prior to sampling. The water source from the local aquifer is well-buffered; hence, sodium bicarbonate was not needed, and pH was checked with each batch of anesthetic used to ensure neutral water. Fish were measured for weight and fork length. One fish per tank was sampled for whole body nutrient analysis (week 8 only). Four fish per tank were sampled for tissues. The whole viscera was removed, weighed, and recorded. Liver samples were taken for FA and transcript expression analysis (week 8 only). Skin was removed from the left side of the fish under the dorsal fin, above the lateral line, and dorsal muscle was then sampled for FA and transcript expression analysis (week 8 only). Tissue samples were placed into 2 mL micro centrifuge tubes, flash frozen in liquid nitrogen and were stored in a −80 °C freezer until they could be shipped to Ryerson University (Toronto, ON) for FA analysis and Memorial University (St. John’s, NL) for transcript expression analysis.

### 2.5. Growth Performance

Parameters to assess growth performance were collected or recorded at week 0 and week 8. Batch weights were recorded at week 4 to ensure fish would reach nearly 300% their initial weight within the 8-week trial period. Fish weight and length were measured on individual fish. Weight gain, condition factor (CF), visceral somatic index (VSI), specific growth rate (SGR), apparent feed intake (AFI) and feed conversion ratio (FCR) were calculated based on the following:

Weight gain (g/fish) = (final weight − initial weight)(1)

CF = body mass/length^3^ (2)

VSI (%) = 100 × (viscera weight/body weight)(3)

SGR (%) = 100 × [ln(final body weight) − ln(initial body weight)]/56 days × 100(4)

AFI (g/fish) = feed consumed per tank (g)/number of fish per tank(5)

FCR = AFI/weight gain(6)

### 2.6. Whole Body Nutrient Composition

Whole rainbow trout were partially thawed and coarsely ground using a bench-top meat grinder and mixed for homogeneity. The sample was frozen and freeze-dried for 72 h and ground into a fine powder using a bench-top homogenizer. Nutrient analysis was completed at the Nova Scotia Department of Agriculture Laboratory Services (Truro, NS, Canada).

### 2.7. Total Lipid and FA Content of Tissues

Total lipid and FA content of liver and muscle sampled from rainbow trout at both weeks 0 and 8 were analyzed. Tissues were freeze-dried and individually ground to a fine powder in liquid nitrogen using a ceramic mortar and pestle (which were washed with soap and water, and then lipid-cleaned three times with 2 mL of chloroform:methanol (2:1; v/v) between each sample), and the resulting powder was subsampled and weighed to the nearest microgram. Total lipid was extracted using a modified Folch method [34]. Briefly, each sample was extracted three times, using 2 mL of chloroform/methanol (2:1; v/v) and then pooled (total 6 mL). Polar impurities were removed by adding 1.6 mL of an aqueous KCl solution (0.9% w/v). The organic layer was removed using a lipid-cleaned glass pipette and pooled. The resulting lipid-containing solvent was concentrated to 2 mL by evaporation with nitrogen gas.

The lipid extract was then prepared for gas chromatography (GC) by derivatizing into fatty acid methyl esters (FAME) using the Hildich reagent (1.5 H_2_SO_4_: 100 anhydrous MeOH) as the catalyst [35]. Reagents were added in the proportion of 1.5 mL reagent per 4–16 mg of lipid. Samples were heated at 90 °C for 90 min and vortexed halfway through the derivatization reaction. The FAME were extracted twice using hexane: diethyl ether (1:1; v/v), then dried under a gentle stream of nitrogen. The dry FAME extract was re-dissolved in hexane and individual FAME were separated using a GC (Shimadzu-2010 Plus, Nakagyo-ku, Kyoto, Japan) equipped with an SP-2560 column (capillary, highly polar, fused silica column, 100 m × 0.25 mm ID × 20 µm thick film; Sigma-Aldrich, St. Louis, MO, USA). All solvents used in the extraction and FAME derivatization procedures were of high purity HPLC grade (>99%). FAME in samples were identified by comparison of their retention times with a known standard (GLC-463 reference standard; Nu-chek Prep, Inc., Waterville, MN, USA) and quantified with a 5-point calibration curve using this same standard. A known concentration of 5 alpha-cholestane (C8003, Sigma-Aldrich, St. Louis, MO, USA) was added to each sample prior to extraction to act as the internal standard to estimate extraction and instrument recovery efficiency.

### 2.8. RNA Preparation

Individual fish in the qPCR study were the same individuals that were analyzed for FA content at week 8. For the 35 liver samples, ~100 mg tissue was added to a 1.5 mL RNase-free tube containing 400 µL of TRIzol reagent (Invitrogen/Thermo Fisher Scientific, Burlington, ON, Canada) and homogenized using a motorized Kontes RNase-Free Pellet Pestle Grinder (Kimble Chase, Vineland, NJ, USA). An additional 400 µL of TRIzol was added and mixed by pipetting. For the 36 muscle samples, ~100 mg tissue was added to a 2 mL RNase-free tube containing 400 µL of TRIzol and a 5 mm stainless steel bead (QIAGEN, Mississauga, ON, Canada), and then homogenized using a TissueLyser (QIAGEN) set at a frequency (1/s) of 25 for 2.5 min. An additional 400 µL of TRIzol was added to the tube and the homogenization step was repeated. Thereafter, for both tissues, the homogenates were frozen on dry ice and stored at −80 °C. They were further disrupted by thawing them on wet ice and then passing them through QIAshredder (QIAGEN) spin columns following the manufacturer’s instructions. Next, 200 µL of TRIzol was added to each sample to make a total homogenate volume of ~1 mL. The TRIzol total RNA extractions were then completed following the manufacturer’s instructions.

The TRIzol-extracted liver RNA samples had low A260/230 ratios, which interferes with column purification. Therefore, subsamples (150 µg) were re-extracted using the phenol-chloroform phase separation method. Briefly, to separate the TRIzol-extracted RNA from organic materials, an equal volume of acid phenol:chloroform:isoamyl alcohol (125:24:1) (pH 4.5) (AM9720; Ambion/Thermo Fisher Scientific) was added to each sample, vortexed for 30 s and centrifuged at 16,100× *g* at 4 °C for 20 min. The RNA was then recovered from the aqueous layer from the previous step by precipitation with 0.1 volumes of 3 M sodium acetate (pH 5.5) (AM9740; Ambion/Thermo Fisher Scientific) and 2.5 volumes of anhydrous ethyl alcohol (Commercial Alcohols Inc., Brampton, ON, Canada) at −80 °C for 1 h, followed by centrifugation at 16,100× *g* at 4 °C for 30 min. To wash the resulting RNA pellet, 1 mL of 75% ethanol was added and centrifuged at 16,100× *g* at 4 °C for 20 min. The ethanol was removed, and the RNA pellet was air-dried at room temperature for 10 min and resuspended in nuclease-free water (Invitrogen/Thermo Fisher Scientific).

The acid phenol:chloroform:isoamyl alcohol extracted liver and the TRIzol extracted muscle RNA samples (45 µg total RNA) were then each treated with 6.8 Kunitz units of DNaseI (RNase-Free DNase Set, QIAGEN) with the manufacturer’s buffer (1× final concentration) at room temperature for 10 min to degrade any residual genomic DNA. DNase-treated RNA samples were column-purified using the RNeasy Mini Kit (QIAGEN) following the manufacturer’s instructions. RNA integrity was verified by 1% agarose gel electrophoresis, and RNA purity was assessed by A260/280 and A260/230 NanoDrop UV spectrophotometry for both the pre-cleaned and the column-purified RNA samples. Column-purified liver RNA samples had A260/280 ratios between 2.09 and 2.13 and A260/230 ratios between 1.80 and 2.37, and column-purified muscle RNA samples had A260/280 ratios between 2.07 and 2.14 and A260/230 ratios between 1.80 and 2.34.

### 2.9. Real-Time Quantitative Polymerase Chain Reaction (qPCR)

#### 2.9.1. qPCR Overview

Expression levels of transcripts with functional annotations related to lipid metabolism including elongation (*elovl2*, *elovl5a*, *elovl5b*), desaturation (*fadsd5*, *fadsd6a* and *fadsd6b*), oxidation [*acox1* and *cpt1* (six isoforms/paralogues)] and eicosanoid synthesis (*alox5a* and *cox1a*) were measured in individual liver (*n* = 12 for the MO and MO/CO diets; *n* = 11 for the FO diet) and muscle samples (*n* = 12 per diet) using qPCR. *Cpt1a2a* was not assessed in liver, and *elovl2* and *elovl5b* were not assessed in muscle, as these transcripts were not expressed at levels that could be accurately detected in those tissues.

#### 2.9.2. Primer Design

qPCR primers for the transcripts of interest (TOIs) were designed herein. Primers for the candidate normalizers were designed previously [36]. For *elovl*, *fadsd* and *acox1*, we examined the putative orthologues of the transcripts assessed in Atlantic salmon (*Salmo salar*), as in as in Xue et al., 2015 [37], Katan et al., 2019 [38] and Caballero-Solares et al., 2018 [39], respectively. To obtain all of the available cDNA sequences for a given orthologous gene from rainbow trout, BLASTn searches of the non-redundant nucleotide (nr/nt) and of the expressed sequence tags (EST) databases of NCBI (*Oncorhynchus mykiss* (taxid:8022) sequences only) were performed using the GenBank accession number for the cDNA sequence from Atlantic salmon (in the above mentioned references). Rainbow trout cDNA sequences for *alox5a* (XM_021580837), *cox1a* (based upon the accession number presented in Ishikawa and Herschman 2007 [40]) and for five of the six *cpt1* family members (based upon the accession numbers presented in Morash et al. 2010 [41]) were obtained from GenBank. These sequences were then used to perform BLASTn searches as above to obtain all of the available cDNA sequences for each gene. The additional *cpt1a* family member (*cpt1a2b*) was identified using these BLASTn searches. All sequence data were obtained in July 2020.

A database of all available cDNA sequences for each gene was created using Vector NTI (Vector NTI Advance 11, Life Technologies). Next, for each gene, multiple sequence alignments were performed for its corresponding cDNA sequences using AlignX (Vector NTI Advance 11.5.4). These alignments were used to determine if the sequences were identical, contained single nucleotide polymorphisms (SNPs)/sequencing errors or represented different gene paralogues/isoforms. In the case of SNPs, these areas were avoided when designing primers. In the case of gene paralogues/isoforms, these alignments identified regions where paralogue/isoform-specific qPCR primers could be designed (see below). The GenBank accession numbers of the sequences used for primer design are presented in Table 4.

Primers were mostly designed using Primer3 ([42,43,44] however, in the case of the gene paralogues/isoforms, some were hand-designed in paralogue/isoform-specific areas to ensure specificity. With the exception of the reverse primer for *fadsd6a* (located in the 3′ untranslated region), all primers are located in the coding sequences. In the case of gene paralogues/isoforms, primers were designed in an area with ≥3 bp difference between them to ensure specificity. The amplicon size range was between 94–155 bp. The sequences, amplicon sizes and efficiencies for all primer pairs used in the qPCR analyses are presented in Table 4.

#### 2.9.3. cDNA Synthesis and qPCR Parameters

First-strand cDNA templates for qPCR were synthesized in 20 μL reactions from 1 μg of DNaseI-treated, column-purified total RNA using random primers (250 ng; Invitrogen/Thermo Fisher Scientific), dNTPs (0.5 mM final concentration; Invitrogen/Thermo Fisher Scientific), M-MLV reverse transcriptase (200 U; Invitrogen/Thermo Fisher Scientific) with the manufacturer’s first strand buffer (1× final concentration) and DTT (10 mM final concentration) at 37 °C for 50 min.

PCR amplifications were performed in 13 μL reactions using 1× Power SYBR Green PCR Master Mix (Applied Biosystems/Thermo Fisher Scientific), 50 nM of both the forward and reverse primers, and the indicated cDNA quantity (see below). Amplifications were performed using the QuantStudio 6 Flex Real Time PCR system (384-well format) (Applied Biosystems/Thermo Fisher Scientific). The real-time analysis program consisted of 1 cycle of 50 °C for 2 min, 1 cycle of 95 °C for 10 min and 40 cycles of 95 °C for 15 s and 60 °C for 1 min, with fluorescence detection at the end of each 60 °C step and was followed by dissociation curve analysis.

#### 2.9.4. Primer Quality Assurance Testing

Each primer pair was quality-tested separately for both liver and muscle. Quality testing ensured that a single product was amplified (dissociation curve analysis) and that there was no primer-dimer present in the no-template control. Amplicons were electrophoretically separated on 2% agarose gels and compared with a 1 kb plus ladder (Invitrogen/Thermo Fisher Scientific) to verify that the correct size fragment was being amplified. Amplification efficiencies were calculated for cDNA pools generated for each tissue. Briefly, cDNAs were synthesized for one individual RNA sample from each of the nine tanks (*n* = 3 per diet) in the study and then pooled (with an equal quantity of cDNA to a given pool). Standard curves then were generated using a 5-point 1:3 dilution series starting with cDNA representing 10 ng of input total RNA. The efficiencies for both liver and muscle are reported in Table 4.

#### 2.9.5. Endogenous Control (Normalizer) Selection

Endogenous control selection was performed as described below for both liver and muscle separately. Expression levels of the TOIs were normalized to transcript levels of two endogenous controls. These endogenous controls were selected from six candidate normalizers (60S ribosomal protein L32 (*rpl32*; CF752566), ATP-binding cassette sub-family F member 2 (*abcf2*; CA383423), β-actin (*actb*; AF157514), elongation factor 1-alpha, oocyte form (*ef1a*; CF752140), eukaryotic translation initiation factor 3 subunit D (*etif3d*; CU070663) and polyadenylate-binding protein cytoplasmic 1 (*pabpc1*; CA355003)) [36]. Briefly, the fluorescence threshold cycle (C_T_) values of 24 samples (i.e., eight samples from each of the three diets) were measured (in duplicate) for each of these transcripts using cDNA representing 5 ng of input total RNA, and then analyzed using geNorm [45]. Based on this analysis, *ef1a* (geNorm M = 0.275) and *actb* (geNorm M = 0.295) were selected as the two endogenous controls for liver; *actb* (geNorm M = 0.275) and *pabpc1* (geNorm M = 0.300) were selected as the two endogenous controls for muscle.

#### 2.9.6. Experimental qPCR Analyses

In the experimental qPCR analyses, cDNA representing 5 ng of input RNA was used as template in the PCR reactions. On each plate, for every sample, the TOIs and endogenous controls were tested in triplicate and a no-template control was included. Since expression levels of a given TOI were assessed across three (Study 1 and 2) or two (Study 3) plates, a plate linker sample (i.e., a sample that was run on all plates in a given study) was also included to ensure there was no plate-to-plate variability. The relative quantity (RQ) of each transcript was determined using the QuantStudio Real Time PCR Software (version 1.3) (Applied Biosystems/Thermo Fisher Scientific) relative quantification study application, with normalization to both *ef1a* and *actb* (liver) or *actb* and *pabpc1* (muscle) transcript levels, and with amplification efficiencies incorporated. For each TOI, the sample with the lowest normalized expression (mRNA) level was set as the calibrator sample (i.e., assigned an RQ value = 1.0).

### 2.10. Statistical Analysis

Growth performance results were analyzed by ANOVA using the general linear model in Minitab 19 Statistical Software. For measurements on individual fish (e.g., weight, length, VSI, CF), a two-level nested ANOVA was used to analyze growth data. This model was designed to test the effect of diet treatment (fixed factor) on the growth performance (response variable) and nested fish individuals (random factor) within tanks, to remove variability among fish within tanks, while also testing for effects of individual tanks [46]. For measurements that were based on tank means, fish individuals were not independent (e.g., weight gain, SGR, AFI, FCR), a one-way ANOVA was conducted to test the effect of diet. Tukey HSD post-hoc tests (*p* < 0.05 significance level) were applied to assess differences among treatments.

For lipid and FA content of liver and muscle, a one-way ANOVA was used to detect treatment differences, followed by a Tukey post-hoc test for multiple comparisons (*p* < 0.05 significance level). Multivariate analyses, including permutational multivariate analysis of variance (PERMANOVA) and Principal Coordinates Analysis (PCoA) were used to determine whole fatty acid profile changes in individual fish tissues among treatments. Vectors were included in the plot that showed correlations >0.75 (Pearson correlation). The non-metric Bray–Curtis dissimilarity statistic was used to quantify the compositional dissimilarity between samples in the PCO plot [47]. PERMANOVA analyses were performed on resemblance matrices, which were built upon the Bray–Curtis similarity matrix. Multivariate statistics (i.e., PCoA and PERMANOVA) were performed using Primer 7 with the PERMANOVA+ add on package (Primer-E version 7, Aukland, New Zealand).

Transcript expression data were log_10_ transformed and one-way ANOVA followed by Tukey’s B post-hoc test, were used to assess expression levels of a given TOI among the three treatments. In all cases, *p* < 0.05 was considered statistically significant and treatments are referred to as “different” only if a statistical difference was found, unless otherwise stated. All data are expressed as mean ± standard deviation (SD).

To relate transcript RQ data with FA data, PCoA was used to provide a representation of FA and transcript expression among treatments at week 8, and PERMANOVA was performed to study the strength and significance level of variation due to diet. Pearson correlation analyses were also used to relate transcript expression RQ data with tissue FA data. Targeted transcripts from this study in liver and muscle were compared with relevant n-3 and n-6 FA (DHA, EPA, arachidonic acid (ARA; 20:4n-6), ALA, LNA, total n-3, total n-6, 18:4n-3, 20:3n-3, 22:5n-3, 18:3n-6, 20:3n-6) in liver and muscle, as well as in the diet. Correlations were performed using SigmaPlot 14.0 (Systat Software, Inc., San Jose, CA, USA).

## 3. Results

### 3.1. Growth Performance

Three mortalities were observed during this experiment. Mortalities of fish occurred during the week prior to final sampling in which fluctuations in temperature resulted in a temperature drop over an hourly period from 14.0 to 11 °C. During this time, the oxygen concentration in the tank system dropped from >100% saturation to 70%. This may have resulted in unanticipated stress on fish resulting in mortality.

Dietary MO inclusion had a significant impact on growth performance (Table 5). Trout fed the FO diet had a longer fork length than trout fed the MO/CO diet (*p* = 0.009; Table 5). Initial CF was higher in trout in the FO treatment compared to trout in the MO/CO treatment; however, this measurement was taken prior to feeding experimental diets, so a treatment effect was not observed at this time. There were no significant differences among treatments for the following measurements: initial weight (*p* = 0.419), final weight (*p* = 0.064), weight gain (*p* = 0.135), initial length (*p* = 0.356), final CF (*p* = 0.623), initial VSI (*p* = 0.552), final VSI (*p* = 0. 169), SGR (*p* = 0.225), AFI (*p* = 0.107), and FCR (*p* = 0.467) (Table 5).

There were no significant differences in the whole-body nutrient content of rainbow trout among treatments for crude protein, total fat, ash, calcium, potassium, magnesium, phosphorus, sodium, and zinc (Table 5).

### 3.2. Fatty Acid Content of the Liver

LNA, ALA, DHA, total PUFA, total n-3, total n-6, and the sum of EPA and DHA were higher in trout fed the MO/CO diet compared to trout fed either the FO or MO diet (*p* < 0.001; Table 6). EPA was higher in trout fed the FO diet compared to trout fed the MO and MO/CO diets (*p* < 0.001). Both EPA and DHA were lower in all treatments after 8 weeks compared to the initial measurement. Total MUFA was higher in liver of trout fed the MO/CO diet compared to the MO diet (*p* = 0.034); however, there was no difference in total MUFA between trout fed the MO/CO diet and the FO diet, or between trout fed the MO diet and FO diet. The n-3/n-6 ratio was highest in trout fed both the FO and MO diets compared to trout fed the MO/CO diet (*p* = 0.003). Total lipid (wet and dry %) was not different among treatments in liver; however, total lipid (wet and dry weight) was higher in trout fed the MO/CO diet when compared to trout fed either the FO or MO diet (*p* < 0.001). After 8 weeks, total lipid stored in the liver decreased in trout fed all treatments compared to the initial measurement (Table 6).

### 3.3. Fatty Acid Content of Muscle

In muscle, LNA was higher in trout fed the MO/CO diet compared to trout fed the FO diet (*p* = 0.036; Table 7) but was not different compared to trout fed the MO diet. ALA was higher in trout fed the MO/CO diet compared to trout fed the FO and MO diet (*p* < 0.001). EPA was higher trout fed the FO diet than in trout fed both the MO and MO/CO diets (*p* < 0.001). DHA was highest in trout fed the MO diet, followed by the MO/CO diet, and was lowest in the FO diet compared to all treatments (*p* < 0.001). Total PUFA was higher in trout fed the MO/CO diet compared to the FO treatment (*p* = 0.018); however, there was no difference between trout fed the FO and MO diet or between the MO and MO/CO treatments. Total n-3 was higher in trout fed the MO and MO/CO diets compared to trout fed the FO diet (*p*= 0.004). The n-3/n-6 ratio was highest in trout fed the MO diet compared to trout fed either the FO or MO/CO diet (*p* < 0.001). The sum of EPA and DHA was higher in muscle of trout fed the MO diet, followed by trout fed the MO/CO diet, and was lowest in trout fed the FO control (*p* < 0.001). C_22_ PUFA were highest in trout fed the MO diet compared to all treatments, and higher in trout fed the MO/CO diet compared to trout fed the FO diet (*p* < 0.001). C_20_ PUFA were highest in trout fed the FO diet than the other treatments (*p* < 0.001). The EPA/ARA ratio was highest in trout fed the FO diet, followed by trout fed the MO diet, and lowest in trout fed the MO/CO diet (*p* < 0.001). No differences were found in total MUFA (*p* = 0.632) and total n-6 (*p* = 0.058) in the muscle. Total lipid (wet and dry weight) in trout muscle was not different among treatments; however, this was higher in all treatments at week 8 compared to the initial measurement (Table 7).

### 3.4. Multivariate Analyses of Fatty Acid Data 

**Figure 1:**
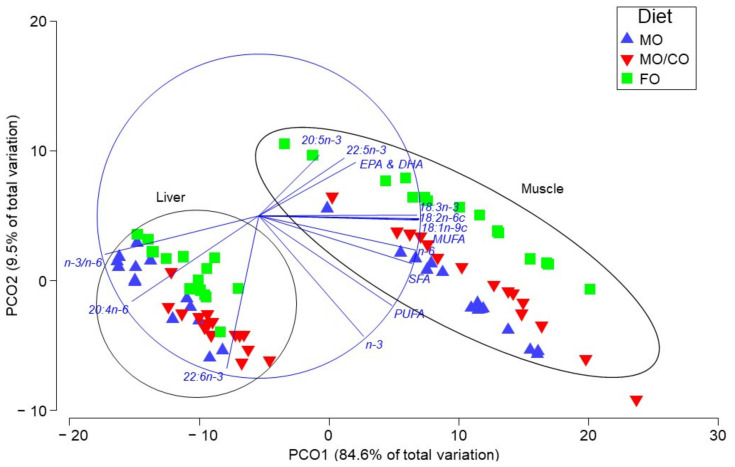
Principal co-ordinate ordination plot of fatty acid profiles of individual rainbow trout tissues (liver and muscle) using a Bray–Curtis similarity matrix, where three diet treatments are represented (FO, MO/CO, MO).

PERMANOVA results indicated that the spatial dispersion of groups was not equivalent, indicating a significant difference in FA content depending on both diet treatment (*p*(perm) < 0.001) and tissue type (*p*(perm) < 0.001); however, diet and tissue factors did not interact (*p*(perm) = 0.324). This is apparent in the PCoA plot (Figure 1), where 94.1% of the variation was accounted for, mainly in PCO1, where a strong distinction among tissue type is present along the PCO1 axis (84.6%) and PCO2 (9.5%) shows distinction among dietary treatments. The FA vectors indicate correlations among liver and DHA, ARA and higher n-3/n-6 ratio, as well as correlations among muscle and EPA, EPA + DHA, MUFA, n-6 and SFA. In addition, the vectors also indicate differences in FA distribution due to treatment. DHA, SFA and MUFA were more strongly correlated with the MO treatment compared to the FO treatment. DHA, n-3, PUFA, and SFA were more correlated with the MO treatment compared to the MO/CO treatment, whereas n-6, MUFA, ALA and LNA were more correlated with the MO/CO treatment compared to the MO treatment. EPA, EPA + DHA, and 22:5n-3 were more correlated with the FO treatment.

### 3.5. Liver Transcript Expression Analysis

**Figure 2:**
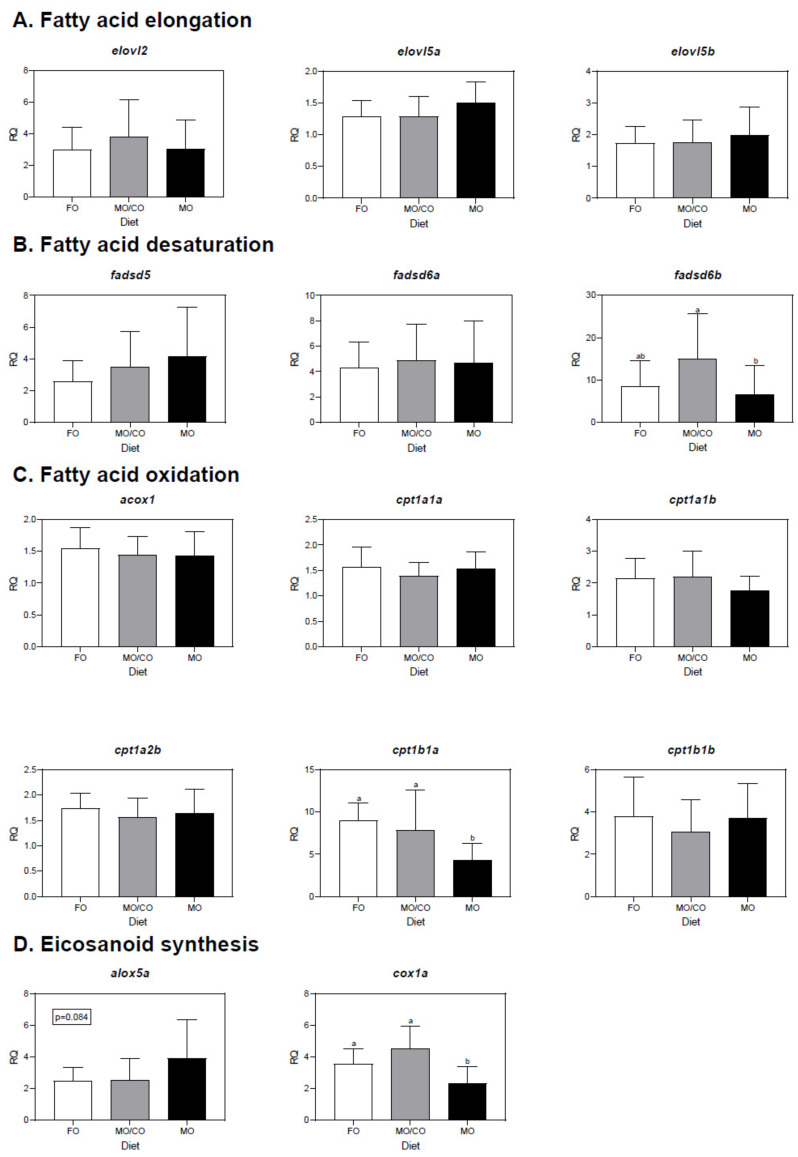
qPCR analyses of transcripts with functional annotations related to lipid metabolism in liver of rainbow trout fed one of three different diets (FO, fish oil; MO/CO, microbial oil/camelina oil blend; MO, microbial oil (*Schizochytrium* sp. T18)). These transcripts include those involved in fatty acid elongation (**A**), fatty acid desaturation (**B**), fatty acid oxidation (**C**) and eicosanoid synthesis (**D**). Transcript levels are presented as mean ± standard deviation relative quantity (RQ) values (i.e., values for the transcript of interest were normalized to both *ef1a* and *actb* transcript levels and were calibrated to the individual with the lowest normalized expression level of that given transcript). Letters indicate Tukey’s HSD groupings. For all treatments, *n* = 3 (11 fish per treatment for the FO diet; 12 fish per treatment for the MO, MO/CO diets) and *p* < 0.05 was considered statistically significant.

Liver transcript expression data can be viewed in Figure 2. For transcripts involved in FA elongation (Figure 2A) or desaturation (Figure 2B), *fadsd6b* had significantly lower expression in trout fed the MO diet compared to trout fed the MO/CO diet (*p* = 0.050). There were no significant differences among treatments for the other transcripts that were either involved in FA desaturation (*fadsd5* and *fadsd6a*) or elongation (*elovl2*, *elovl5a*, *elovl5b*).

For transcripts involved in FA oxidation (Figure 2C), *cpt1b1a* was higher in rainbow trout fed the FO or MO/CO diets compared to trout fed the MO diet (*p* < 0.001; Figure 2). Other transcripts involved in FA oxidation in this study (*acox1*, *cpt1a1a*, *cpt1a1b*, *cpt1a2b*, *cpt1b1b*) were not differentially expressed among treatments.

*Cox1a*, involved in eicosanoid synthesis, was higher in trout fed the FO diet or MO/CO diet compared to the MO diet (*p* < 0.001; Figure 2D). *Alox5a*, also involved in eicosanoid synthesis, was not differentially expressed among treatments (*p* = 0.084).

### 3.6. Muscle Transcript Expression Analysis

**Figure 3:**
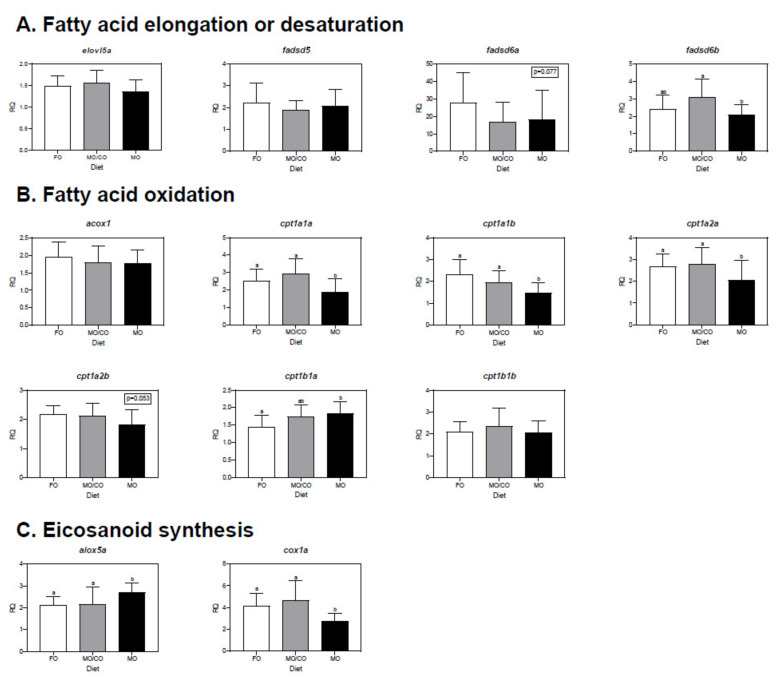
qPCR analyses of transcripts with functional annotations related to lipid metabolism in muscle of rainbow trout fed one of three different diets (FO, fish oil; MO/CO, microbial oil/camelina oil blend; MO, microbial oil (*Schizochytrium* sp. T18)). These transcripts include those involved in fatty acid elongation or desaturation (**A**), fatty acid oxidation (**B**) and eicosanoid synthesis (**C**). Transcript levels are presented as mean ± standard deviation relative quantity (RQ) values (i.e., values for the transcript of interest were normalized to both *actb* and *pabpc1* transcript levels and were calibrated to the individual with the lowest normalized expression level of that given transcript). Letters indicate Tukey’s HSD groupings. For all treatments, *n* = 3 (12 fish per treatment) and *p* < 0.05 was considered statistically significant.

Muscle transcript expression data can be viewed in Figure 3. For transcripts involved in FA elongation (Figure 3A) or desaturation (Figure 3B), *fadsd6b* was higher in trout fed the MO/CO diet compared to trout fed the MO diet (*p* = 0.027); however, *elovl5a*, *fadsd5*, and *fadsd6a* were not differentially expressed among treatments.

For transcripts involved in FA oxidation, *cpt1a1a* (*p* = 0.006), *cpt1a1b* (*p* = 0.003), and *cpt1a2a* (*p* = 0.020) were higher in trout fed the FO and MO/CO diets compared to trout fed the MO diet (Figure 3C). *Cpt1b1a* was higher in trout fed the MO diet compared to trout fed the FO diet (*p* = 0.018). *Acox1*, *cpt1a2b*, *cpt1b1b* were not differentially expressed among treatments.

For transcripts involved in eicosanoid synthesis, *alox5a* was higher in trout fed the MO diet compared to trout fed the FO and MO/CO diets (*p* = 0.018; Figure 3C). *Cox1a* was higher in trout fed the FO and MO/CO diets compared to rainbow trout fed the MO diet (*p* = 0.009).

### 3.7. Multivariate Statistics and Correlations of Fatty Acid and Transcript Datat

PERMANOVA revealed that diet was a significant factor for both liver (*p*(perm) = 0.006) and muscle (*p*(perm) = 0.017) when considering FA and RQ data. In liver, 81.3% of the variation was accounted for in PCO1, with 8.4% of the variation in PCO2 (Supplementary Figure S1). Vectors indicated that DHA, *alox5a*, *cpt1b1b*, *fadsd5*, and *elovl5a* were more strongly correlated with trout fed the MO and MO/CO diets. The remaining transcripts, as well as EPA, EPA + DHA, total PUFA, MUFA, and SFA were more correlated with trout fed the FO diet. In muscle, 84.4% of the variation was accounted for in PCO1, with 8.2% of the variation in PCO2 (Supplementary Figure S2). Vectors indicated that n-3/n-6, DHA, *alox5a*, *cpt1b1a* were strongly correlated with trout fed the MO and MO/CO treatments. All remaining targeted transcripts, as well as EPA, EPA + DHA, total PUFA, MUFA and SFA were strongly correlated with trout in the FO treatment.

Correlation analyses revealed a significant positive relationship between EPA and *cpt1b1a* (R = 0.420; *p* = 0.0120) in liver; however, there were no other significant relationships between transcript expression and FA content in liver (Supplementary Table S1). In muscle, *fadsd6b* showed a significant positive relationship with ALA (R = 0.350; *p* = 0.0362) and with 20:3n-3 (R = 0.398; *p* = 0.0162). Transcript expression levels of *cpt1a1b* showed a significant negative relationship with DHA (R = −0.455; *p* = 0.005) and a significant positive relationship with EPA (R = 0.405; *p* = 0.0143). Transcript expression levels of *cpt1b1a* were significantly positively correlated with DHA (R = 0.418; *p* = 0.0113) and negatively correlated with EPA (R = −0.458; *p* = 0.00494). The remaining relationships among selected transcripts and FA were not significant in muscle (Supplementary Table S1). Dietary DHA was negatively correlated with *cpt1b1a* (R = −0.632; *p* < 0.001) and *cox1a* (R = −0.484; *p* = 0.003) in liver (Supplementary Table S2). Dietary EPA (R = 0.530; *p* = 0.001) and ARA (R = 0.486; *p* = =0.003) were positively correlated with *cpt1b1a* in liver. Dietary DHA was negatively correlated with *cpt1a1a* (R = −0.413; *p* = 0.012), *cpt1a1b* (R = −0.558; *p* < 0.001), *cpt1a2a* (R = −0.432; *p* = 0.009), *cpt1a2b* (R = −0.408; *p* = 0.014) and *cox1a* (−0.440; *p* = 0.007) in muscle and was positively correlated with *cpt1b1a* (R = 0.409; *p* = 0.013) and *alox5a* (R = 0.407; *p* = 0.014; Supplementary Table S2). Dietary EPA was positively correlated with *cpt1a1b* (R = 0.468; *p* = 0.004) and negatively correlated with *cpt1b1a* (−0.411; *p* = 0.013) in muscle. Dietary ARA was also positively correlated with *cpt1a1b* (R = 0.431; *p* = 0.009) and negatively correlated with *cpt1b1a* (R = −0.395; *p* = 0.017) in muscle. Dietary ALA was positively correlated with *fadsd6b* (R = 0.393; *p* = 0.018) in muscle (Supplementary Table S2).

## 4. Discussion

Growth in the aquaculture sector faces several challenges related to the sustainable production of n-3 LC-PUFA to meet both farmed fish and consumer nutritional demands. The application of novel microbial oils, such as *Schizochytrium* sp., in aquafeeds presents an opportunity to eliminate or reduce dependence on wild-harvested FO as a principal source of n-3 LC-PUFA. Previous research on post-smolt Atlantic salmon [14,19,20], Nile tilapia (*Oreochromis niloticus* [48]), and rainbow trout [29] have demonstrated the applicability of various strains of *Schizochytrium* sp. oil as a feasible dietary lipid alternative to FO. The present study evaluated the application of high-DHA *Schizochytrium* sp. (T18) oil, as a dietary lipid source in diets for rainbow trout, as a complete replacement for FO. DHA quantities within the MO/CO and MO diets were substantially higher than the FO diet, indicating that the *Schizochytrium* sp. The (T18) oil used in this study can be supplied at both lower (75 g/kg) and higher (100 g/kg) concentrations to provide DHA equivalent to that of FO, although minimal EPA was supplied.

### 4.1. Impact on Growth Performance and Whole Body Nutrient Content

Overall, the substitution of FO with MO in diets for juvenile rainbow trout generally had no effect on growth performance, FCR, or whole-body nutrient content. Trout fed the MO/CO diet were slightly shorter in fork length compared to trout fed the FO diet, though there was no difference in body size in terms of weight or CF due to this difference in length. Feed intake and feed conversion ratio were not significantly different among treatments. However, it is not surprising that dietary inclusion of MO did not impact growth performance, since both EPA and DHA were provided in more than sufficient amounts to meet dietary requirements (with EPA supplied from the lipid content in fish meal). Further, it has also been shown that dietary n-3 LC-PUFA play an important role in feeding behavior and feed intake of juvenile rainbow trout [49], while it has actually been demonstrated that fatty acid sensing mechanisms in rainbow trout hypothalamus are responsive to changes in the levels of long-chain fatty acids (as reviewed by Soengas 2021 [50]). This suggests that replacement of FO with MO resulted in a dietary FA composition which was palatable and consequently resulted in similar feed intake and growth.

The results of the present study were also consistent with previous findings reported by Santigosa et al. (2020) [29], where juvenile rainbow trout were provided with *Schizochytrium* sp. oil at inclusion rates ranging from 2.5, 3, 5, and 10% of the diet, which showed no adverse effects on growth performance. *Schizochytrium* sp. oil was also included at 13% of the diet for Atlantic salmon and showed no significant difference compared to the FO control treatment in terms of growth performance [28]. Similar findings were reported by Tibbets et al., (2020) [14] for juvenile Atlantic salmon, where using *Schizochytrium* sp. (T18) oil as a complete replacement for FO did not result in significant treatment effects in growth performance compared to the FO control. Together, these findings suggest that the use of *Schizochytrium* sp. in diets for rainbow trout and Atlantic salmon (both parr and post-smolts) show no impacts on growth performance in comparison to diets containing FO as the primary lipid source [14,20,28,29].

Dietary inclusion of dried whole-cell *Schizochytrium* sp. as a FM and FO replacement has also been assessed for its effects on growth performance and digestibility. Sarker et al. (2016) [48] evaluated the inclusion of dried whole-cell *Schizochytrium* sp. in replacing FO at 25%, 50%, 75%, and 100% inclusion in diets for Nile tilapia over 84 d. Tilapia fed diets containing dried whole-cell *Schizochytrium* sp. were found to have increased final weight, increased weight gain, and reduced FCR [48]. In contrast, the application of dried whole-cell *Schizochytrium* sp. in diets of Atlantic salmon had no significant impact on growth performance [51]. Dried whole-cell *Schizochytrium* sp. was also found to be highly digestible by Atlantic salmon when included at 30% of the diet, with PUFA and DHA > 95% digestible and were significantly more digested in the *Schizochytrium* sp. diet compared to the control diet [27]. Another study tested the digestibility of whole-cell *Schizochytrium* sp. by juvenile rainbow trout and found that dry matter, crude protein, total fat, energy, and fatty acids were all >85% digestible [52]. Longer-term growth performance and fish health studies would be required to evaluate the relative merits of *Schizochytrium* sp. whole cell biomass vs. extracted oil.

### 4.2. Impact on Liver FA Content

The liver is a primary site of lipid metabolism and involves activities such as lipogenesis, oxidation, and biosynthesis of LC-PUFA [53]. As such, the liver is considered an indicator of change for lipid and fatty acid metabolism. In the present study, the inclusion of high DHA *Schizochytrium* sp. oil in diets for trout significantly affected liver FA profiles. EPA in liver was lower in trout that were fed either the MO or MO/CO diets compared to the FO diet. Considering the low levels of EPA in *Schizochytrium* sp. oil used in this study, the reduced amount of EPA stored in the liver in these treatments is expected. DHA was highest in trout fed the MO/CO diet compared to trout fed either the FO or MO diet. This is an interesting result because it contrasts with what was observed in the muscle and does not match dietary DHA levels (i.e., the MO diet had the highest amount of DHA). It is also interesting that liver DHA amounts were lower in all treatments after 8 weeks compared to the initial measurement, which was not the case in muscle. Future studies should also relate this to the hepatosomatic index. While the conservation of DHA in the liver was expected (i.e., DHA sparing [54]), as liver is relatively high in phospholipid (PL) compared to muscle (which is high in triacylglycerol; TAG) and is more resistant to dietary change, it was unexpected that trout fed the MO/CO diet stored the most DHA. This may be due to the selective retention of DHA, as a result of higher levels of dietary C_18_ PUFA in the MO/CO diet. Selective retention or sparing of DHA has been previously documented in salmon and rainbow trout that were fed vegetable-oil-based diets (e.g., flaxseed, sunflower, camelina oils) with high levels of dietary C_18_ PUFA [55]. Additionally, the lack of evidence of additional EPA storage in the liver, suggests that retro-conversion of DHA to EPA did not occur to a significant extent, (probably because it was not required given dietary levels). This finding was also suggested in the study by Wei et al. (2021) [20] with Atlantic salmon fed diets containing oil from *Schizochytrium* sp. (T18). It is unknown whether low reserves of EPA in the liver could eventually have consequences for fish health, e.g., if biosynthesis from ALA or retro-conversion from DHA cannot keep up with demand.

### 4.3. Impact on Muscle FA Content

The replacement of FO by high-DHA *Schizochytrium* sp. (T18) oil (at both lower and higher inclusion levels) significantly influenced FA content in trout muscle, and generally, muscle was reflective of the FA composition of the diet due to MO inclusion. Similarly, in the Miller et al. (2007) [28] study, the major difference between treatments was in the FA profile of the salmon muscle, particularly the n-3 and n-6 LC-PUFA content. Furthermore, in the present study, most notable was the higher DHA content in trout fed diets containing MO (at both higher and lower inclusion levels) compared to trout fed the FO diet. This result was also reported by Wei et al. (2021) [20] in Atlantic salmon muscle. Higher amounts of DHA stored in muscle is expected due to the significantly higher content of DHA in the MO and MO/CO diets compared to the FO diet. On the other hand, EPA was much lower in the MO and MO/CO diets compared to the FO diet, and consequently, significantly lower levels of EPA were stored in trout muscle that were fed the MO-containing diets compared to trout fed the FO diet. Previous studies have reported similar findings when *Schizochytrium* sp. oil was tested as a FO alternative in diets for rainbow trout and Atlantic salmon [28,29,56,57], as well as *Schizochytrium* sp. whole-cell biomass in rainbow trout [58].

ALA and LNA stored in muscle were highest in trout fed the MO/CO diet compared to trout fed either the FO or MO diet. The increase in these C_18_ PUFA in trout fed the MO/CO diet is attributed to the dietary CO inclusion, which is characteristically high in these FA, and is also known to be accumulated in the muscle of fish when fed diets containing CO, such as rainbow trout [33], Atlantic salmon [59], tilapia [60], and gilthead seabream (*Sparus aurata* [61]). Lower dietary EPA content was reflected in the muscle tissue, with EPA ~3x lower in trout fed the MO and MO/CO diets compared to the FO diet. Given the lower EPA amount in the MO diets, it was hypothesized that this may induce biosynthetic production of EPA from ALA to compensate for lower dietary EPA. However, it is likely that the MO diets still supplied enough EPA, since the diet provided at least 1.5 μg/mg EPA (from FM) and EPA in muscle was stored at >3 μg/mg. Despite differences in ALA, EPA, and DHA accumulation, no significant differences were noted in total lipid within trout fed any of the experimental diets. However, since EPA is a precursor to anti-inflammatory classes of eicosanoids, which contributes to the immune response and fish health [56], subsequent research must be completed to observe the impact on reduced dietary EPA available within fish relative to growth, and subsequent potential impacts on immune system function and response.

Total n-3 PUFA content was higher in trout fed the MO and the MO/CO diet compared to the FO control. As a result, the n-3/n-6 ratio was highest in trout fed the MO diet compared to any dietary treatment. Although these findings contrast with Wei et al. (2021) [20], where total n-3 amounts were higher in muscle of salmon fed the FO diet compared to salmon fed with both low and high concentrations of *Schizochytrium* sp. (T18) oil, it depends on the FO inclusion level. In the Wei at al. (2021) [20] study, FO was included at 20% of the diet, compared to 10% inclusion in the current study. From a human health perspective, consuming foods with a higher total n-3 content (i.e., rainbow trout) and higher n-3/n-6 ratio is beneficial [62]. Further, rainbow trout fillets from both the MO and MO/CO treatments contain EPA + DHA amounts that meet the recommended daily requirement for EPA + DHA intake of 250 mg, as suggested by both the World Health Organization (WHO, 2008 [63]) and the Global Organisation for EPA and DHA (GOED, 2014 [64]). The recommended serving of fish is 100 g of cooked fish [65]. The sum of EPA + DHA (wet weight) in 100 g fillet of trout fed FO, MO/CO, and MO is 799 mg, 1008 mg, and 1252 mg, respectively. As such, the consumption of one-100 g serving of fillets from rainbow trout that were fed either MO or MO/CO is more than sufficient to meet the daily requirement (250 mg). In fact, for trout fed the MO diet, consumers need only eat 1.4 servings (140 g fillet) per week to achieve the total weekly EPA + DHA requirement.

### 4.4. Multivariate Analysis of FA profiles

Based on the spatial dispersion of datapoints representing the FA profile of each trout (see Figure 1), both tissue type and diet contributed to the variation of fatty acid profiles of trout in this study, although they were not interacting factors. Most of the variation was attributed to FA differences in liver vs. muscle, regardless of treatment, suggesting that tissue type was a more defining factor in FA composition than diet. Muscle tissue in salmonids species is known to be receptive to dietary change (due to the high TAG, which stores FA from the diet [66]), whereas liver (high in PL) does not store dietary FA as readily with membrane-bound FA [53].

### 4.5. Transcript Expression in Liver

Regarding LC-PUFA biosynthesis, *fadsd6b* was >2-fold higher in trout fed the MO/CO diet compared to trout fed the MO diet. The delta-6 desaturase enzyme is the first step toward, desaturating ALA to 18:4n-3, and then also desaturates 24:5n-3 to 24:6n-3, before oxidation (and chain shortening) to synthesize DHA. In this study, for trout fed the MO/CO diet, either scenario is possible; although the former is more likely because even the MO/CO diet contained sufficient DHA, it was not necessary to synthesize more. Trout fed the MO/CO diet had higher liver DHA, higher ALA and similar EPA levels compared to trout fed the MO diet. The high ALA coupled with low EPA in these fish may have triggered biosynthesis from ALA to EPA; therefore, it is more likely that *fadsd6b* was upregulated to work toward biosynthesis from ALA to EPA, rather than the final steps toward DHA synthesis (i.e., 24:5n-3 to 24:6n-3). The different results observed between *fadsd6b* and *fadsd6a* (which was not differentially expressed among treatments) are not surprising, since it has been shown that Atlantic salmon show a different pattern of regulation for these paralogous genes in response to a diet that was primarily vegetable-oil-based [67].

Regarding transcript expression related to FA oxidation in liver, *cpt1b1a* (involved in mitochondrial β-oxidation and in overall FA turnover in liver) was more highly expressed in trout fed the FO and MO/CO diets compared to trout fed the MO diet. Oxidation of FA in the MO/CO diet may suggest that the C_18_ PUFA (which were highest in the MO/CO diet compared to the other diets) were readily used from dietary CO as a substrate for β-oxidation. On the other hand, the oxidation of FA from the FO diet may suggest that ample essential FA were provided and were substrates for β-oxidation. Trout that were fed the MO diet contained lower EPA and lower C_18_ PUFA, so it is possible that *cpt1b1a* was not upregulated because there was no excessive dietary FA available for β-oxidation. The upregulation of *cpt1b1a* might also contribute to a lipid-lowering effect, which was observed in rainbow trout fed diets containing the macroalgae, *Saccharina latissima*, and was significantly negatively correlated with the hepatosomatic index and final body weight in a study by Ferreira et al. (2020) [68]. In the present study, correlation analysis revealed that *cpt1b1a* was significantly positively correlated with EPA in liver. In the FO and MO/CO diets, more dietary EPA may have been substrate for β-oxidation compared to DHA when it was bioavailable, which has been suggested previously in different fish species, including salmon [69,70,71]. Several studies have demonstrated changes in β-oxidation both at the enzymatic and gene expression level in salmonid species when there is a shift in substrate availability due to replacing dietary FO with alternative oils [72,73,74] and upregulation of *cpt1b1a* by dietary lipid has been demonstrated in previous observations in rainbow trout [41,75].

*Cox1a*, involved in eicosanoid synthesis, was more highly expressed in trout fed the FO diet and MO/CO diet compared to the MO diet. This may be because ample EPA that was provided in the FO diet and the MO/CO diet was a substrate for eicosanoid production, whereas trout fed the MO diet had very low amounts of EPA. The dietary n-3/n-6 ratio matters because the cyclooxygenase (COX) and lipoxygenase (LOX) enzymes compete for ARA and EPA for the production of eicosanoids (prostaglandins, leukotrienes; [76]. Although the liver EPA/ARA ratio in trout fed the MO diet (0.4) was much lower than the FO diet (1.5), the n-3/n-6 ratio in the MO diet was much higher. However, it has been shown that DHA can lower *cox2* transcript expression in the human brain [77]; therefore, it is possible that DHA in the MO diet led to a decrease in *cox1a* transcript expression; however, future research is needed to identify the mechanism. For example, it will be important to assess *alox12* and *alox15* in this pathway, as well as the protein level or oxylipin levels. However, there is not a straightforward relationship between the amount of precursor FA in the cell membranes and eicosanoid production [76]. The biological effects of eicosanoids may depend more on their ratios than total amounts. For example, Sissener et al. (2020) [76] found that there was a gradient towards dominance of the n-6 derived eicosanoids with increasing dietary n-6 for the LOX pathway in liver, but not for the COX pathway [76]. Katan et al. (2020) [78] found that *cox1* expression could result in production of anti-inflammatory prostaglandins in Atlantic salmon head kidney at the constitutive level, and that differences among treatments in head kidney EPA/ARA may have altered *cox1* transcription. This also appears to be the case in trout liver in the present study. It is well known that changes in cell membrane EPA/ARA could alter eicosanoid production and the inflammatory response in vertebrates [79,80] however, eicosanoid analysis was beyond the objectives of this study.

### 4.6. Transcript Expression in Muscle

Regarding LC-PUFA biosynthesis, *fadsd6b* was higher in trout fed the MO/CO diet compared to trout fed the MO diet and was also positively correlated with ALA and 20:3n-3. This suggests biosynthesis toward n-3 LC-PUFA occurred, since the MO/CO diet provided lower amounts of DHA than the MO diet. In terms of FA oxidation, *cpt1a1a*, *cpt1a1b*, *cpt1a2a* were highly expressed in trout fed the FO or MO/CO diets compared to trout fed the MO diet. Conversely, *cpt1b1a* was higher in trout fed the MO diet compared to trout fed the FO diet. The opposite pattern of *cpt1* expression between *cpt1a* (downregulated in the MO treatment) compared to *cpt1b* (upregulated in the MO treatment) suggests there may be differences in function between the *cpt1a* and *b* isoforms, the presence of which have both been reported in liver and muscle in rainbow trout [41,75].

For transcripts involved in eicosanoid synthesis, *alox5a* was more highly expressed in trout fed the MO diet compared to trout fed the FO or MO/CO diets. A similar trend was observed in liver, but the difference was not significant. Katan et al. (2020) [78] showed *5loxa* showed positive correlations with both n-3 (i.e., EPA, DHA) and n-6 (ARA) LC-PUFA in Atlantic salmon head kidney. The latter result is also in line with Katan et al., (2019) [38], who reported that hepatic *5loxa* showed a positive relationship with liver EPA + ARA in Atlantic salmon fed different mixtures of plant oils as a FO replacement. Further, a high dietary ARA/EPA ratio appeared to favor increased expression of *5loxa* in Atlantic salmon head kidney [81]. In salmon liver, *5loxa* was correlated negatively with liver EPA and DHA in salmon fed terrestrial-based diets [82]. Again, this may indicate the importance of the ARA/EPA ratio in expression of transcripts involved in eicosanoid synthesis, as well as differences in expression depending on tissue (i.e., head kidney vs. liver) and/or dietary inputs (e.g., protein and lipid sources). The results in the present study suggest it is possible that *alox5a* was more highly expressed in trout fed the MO treatment due to the high levels of DHA stored in the muscle, and although not significantly correlated, may have caused upregulation of *alox5a* as an eicosanoid substrate.

Similar in liver, *cox1a* in muscle was more highly expressed in trout fed the FO and MO/CO diets compared to trout fed the MO diet. This may be because ample EPA that was provided in the FO diet and MO/CO diet led to eicosanoid production from EPA; although muscle EPA was the same in trout fed the MO diet compared to the MO/CO diet.

## 5. Conclusions

High DHA *Schizochytrium* sp. (T18) oil was an effective dietary DHA source in diets for rainbow trout. Inclusion of *Schizochytrium* sp. (T18) oil at high or low levels in the diet (MO and MO/CO diets) resulted in a similar growth performance as seen in trout fed FO; however, muscle and liver FA profiles were impacted due to the diet. Trout fed the MO diets had greater amounts of DHA in their liver and muscle tissues than trout fed the FO diet, and contained lower EPA, but total n-3 was highest in trout fed the highest inclusion level of *Schizochytrium* sp. oil (on account of the high DHA stored). Both high and low inclusion levels of *Schizochytrium* sp. (T18) oil can provide a source of DHA in aquafeeds but does not supply ample EPA. This would require further investigation to evaluate possible impacts on fish health; however, this did not impact fish growth performance over the 8-week study. Future research is necessary to consider *Schizochytrium* sp. oil use in rainbow trout diets for a longer production period, including trout that are closer to market production size.

## Figures and Tables

**Table 1:** FA composition of the microbial oil (MO; *Schizochytrium* sp. T18) used in the study.

FA	MO ^1^ (%)	MO (µg/mg)
14:0	13.1	193.0
15:0	1.5	21.9
16:0	30.7	453.5
16:1n-7c	4.5	66.5
16:1n-7t	<0.1	0.3
17:0	0.3	4.2
17:1n-7	<0.1	0.2
18:0	0.9	13.6
18:1n-9c	0.6	9.0
18:1n-9t	<0.1	0.0
18:1n-12	<0.1	0.0
18:1n-7c	2.8	41.0
18:1n-7t	<0.1	0.0
19:0	<0.1	0.2
18:2n-6 (LNA) ^2^	0.3	4.4
20:0	0.1	0.8
18:3n-6	0.1	1.6
20:1n-15	<0.1	0.0
20:1	<0.1	0.0
20:1n-9	<0.1	0.3
18:3n-3 (ALA) ^3^	0.1	1.2
18:2n-6t	0.1	0.8
18:4n-3	0.2	2.6
20:2n-6	0.0	0.0
22:3n-3	0.1	0.8
22:0	<0.1	0.3
20:3n-6	0.1	1.1
22:1n-9	<0.1	0.5
20:3n-3	<0.1	0.0
20:4n-6 (ARA) ^4^	0.1	1.8
22:2n-6	<0.1	0.0
24:0	0.1	1.6
20:5n-3 (EPA) ^5^	0.7	10.5
24:1n-9	<0.1	0.0
22:4n-6	<0.1	0.2
22:5n-3	0.1	1.8
22:6n-3 (DHA) ^6^	43.4	640.7
ΣSFA ^7^	46.7	689.1
ΣMUFA ^8^	8.2	120.6
ΣPUFA ^9^	45.2	666.7
ΣMUFA ≥ 18C	3.5	51.6
ΣMUFA > 18C	0.1	1.6
ΣC18 PUFA	0.7	10.5
ΣC20 PUFA	0.9	13.4
ΣC22 PUFA	43.6	642.8
ΣEPA & DHA	0.2	651.2
Σn-6	0.7	9.9
Σn-3	44.5	656.8
ΣOdd chain	1.9	28.0
n-3/n-6	67.0	67.0

^1^ Microbial Oil ^2^ Linoleic acid ^3^ Alpha linolenic acid ^4^ Arachidonic acid ^5^ Eicosapentaenoic acid ^6^ Docosahexaenoic acid ^7^ Saturated fatty acid ^8^ Monounsaturated fatty acid ^9^ Polyunsaturated fatty acid.

**Table 2:** Diet formulation and composition (g/kg, as fed basis) of experimental diets fed to rainbow trout.

Ingredient ^1^ (g/kg)	FO	MO/CO	MO
Fish meal	150	150	150
Fish (herring) oil	100	0	0
Microbial oil (MO) ^2^	0	75	100
Camelina oil ^3^	50	75	50
Ground wheat	170	170	170
Empyreal (corn protein concentrate)	120	120	120
Poultry by-product meal	210	210	210
Blood meal	160	160	160
Vitamin and mineral mix ^4^	2	2	2
Dicalcium phosphate	20	20	20
Pigment mix ^5^	2.5	2.5	2.5
Lysine HCl	5	5	5
Choline chloride	10.5	10.5	10.5
**Chemical composition (as fed, g/kg)**			
Dry matter	94.7	95.1	94.9
Crude protein	44.2	44.5	44.6
Total lipid	23.9	24.1	24.2

^1^ All ingredients were supplied and donated by Northeast Nutrition (Truro, NS, Canada) for all experimental diets (FO, fish oil; MO/CO, microbial oil/camelina oil blend; MO, microbial oil) ^2^ MO (*Schizochytrium* sp. T18) was supplied by Mara Renewables (Dartmouth, NS, Canada) ^3^ Commercial grade camelina oil, produced by Smart Earth Seeds (Saskatoon, SK, Canada) ^4^ Vitamin and mineral premix contains (/kg): zinc, 77.5 mg; manganese, 125 mg; iron, 84 mg; copper, 2.5 mg; iodine, 7.5 mg; vitamin A, 5000 IU; vitamin D, 4000 IU; vitamin K, 2 mg; vitamin B12, 0.004 mg; thiamine, 8 mg; riboflavin, 18 mg; pantothenic acid, 40 mg; niacin, 100 mg; folic acid, 4 mg; biotin, 0.6 mg; pyridoxine, 15 mg; inositol, 100 mg; ethoxyquin, 42 mg; wheat shorts, 1372 mg. ^5^ Pigment mix contains (/kg): selenium, 0.220 mg; vitamin E, 250 IU; vitamin C, 200 mg; astaxanthin, 60 mg; wheat shorts, 1988 mg.

**Table 3:** FA content (μg/mg, dry weight; FAME % in parentheses) of experimental diets fed to rainbow trout ^1^.

FA	FO	MO/CO	MO	F-Value	*p*-Value
14:0	10.8 ± 0.5 ^b^ (4.3)	12.3 ± 0.9 ^b^ (5.0)	18.3 ± 1.6 ^a^ (6.8)	40.25	0.000
16:0	45.7 ± 3.0 ^b^ (18.1)	49.3 ± 5.0 ^b^ (20.1)	61.3 ± 4.2 ^a^ (22.8)	11.77	0.008
16:1n-7	13.2 ± 0.5 ^a^ (5.2)	7.8 ± 0.7 ^c^ (3.2)	9.5 ± 0.8 ^b^ (3.5)	49.08	0.000
18:0	9.8 ± 0.5 ^a^ (3.9)	6.8 ± 0.6 ^b^ (2.8)	6.6 ± 0.5 ^b^ (2.5)	30.76	0.001
18:1n-9	41.3 ± 1.4 ^a^ (16.4)	36.0 ± 3.5 ^ab^ (14.7)	31.5 ± 2.6 ^b^ (11.7)	10.28	0.012
18:2n-6 (LNA) ^2^	27.3 ± 0.9 (10.8)	31.9 ± 2.8 (13.1)	29.0 ± 2.4 (10.9)	3.33	0.107
18:3n-3 (ALA) ^3^	25.0 ± 0.8 ^b^ (9.9)	31.1 ± 1.5 ^a^ (12.8)	25.1 ± 2.5 ^b^ (9.4)	11.74	0.008
20:1n-9	13.0 ± 0.3 ^b^ (5.2)	15.6 ± 1.1 ^a^ (6.4)	12.6 ± 1.1 ^b^ (4.7)	9.61	0.013
20:4n-6 (ARA) ^4^	1.3 ± 0.0 ^a^ (0.5)	0.6 ± 0.0 ^b^ (0.2)	0.7 ± 0.1 ^b^ (0.3)	190.94	0.000
20:5n-3 (EPA) ^5^	19.0 ± 0.8 ^a^ (7.5)	1.8 ± 0.1 ^b^ (0.8)	2.1 ± 0.2 ^b^ (0.8)	1229.24	0.000
22:1n-9	2.2 ± 0.0 ^b^ (0.9)	2.8 ± 0.2 ^a^ (1.1)	2.3 ± 0.2 ^b^ (0.8)	9.83	0.013
22:5n-3	2.2 ± 0.1 ^a^ (0.9)	0.3 ± 0.0 ^b^ (0.1)	0.4 ± 0.0 ^b^ (0.1)	1950.49	0.000
22:6n-3 (DHA) ^6^	13.2 ± 0.2 ^c^ (5.2)	28.6 ± 0.8 ^b^ (11.7)	48.9 ± 4.9 ^a^ (18.2)	118.78	0.000
24:1n-9	1.1 ± 0.0 ^a^ (0.4)	0.8 ± 0.1 ^b^ (0.3)	0.7 ± 0.1 ^c^ (0.2)	34.65	0.001
∑SFA ^7^	70.1 ± 4.0 ^b^ (27.8)	72.3 ± 6.7 ^b^ (29.5)	90.8 ± 6.7 ^a^ (33.8)	10.95	0.010
∑MUFA ^8^	86.3 ± 2.4 ^a^ (34.3)	73.6 ± 6.5 ^ab^ (30.1)	68.0 ± 5.9 ^b^ (25.3)	9.72	0.013
∑PUFA ^9^	95.3 ± 2.9 (37.9)	98.3 ± 5.4 (40.3)	109.9 ± 10.2 (40.9)	3.80	0.086
∑n-3	63.6 ± 1.9 ^b^ (25.3)	63.4 ± 2.5 ^b^ (26.0)	78.0 ± 7.8 ^a^ (29.0)	8.97	0.016
∑n-6	31.6 ± 1.0 (12.6)	34.9 ± 3.0 (14.3)	31.9 ± 2.6 (11.9)	1.74	0.254
n-3/n-6	2.0 ± 0.0 ^b^ (2.0)	1.8 ± 0.1 ^b^ (1.8)	2.4 ± 0.1 ^a^ (2.4)	42.82	0.000
Total	251.7 ± 9.1	244.2 ±18.5	268.7 ± 22.5	1.51	0.295

^1^ Data expressed as μg/mg dry weight, values are means (*n* = 3) ± standard deviation. Means with different superscripts indicate significant differences based on Tukey’s post-hoc test following a one-way ANOVA. FO, fish oil; MO/CO, microbial oil/camelina oil blend; MO, microbial oil (*Schizochytrium* sp. T-18). ^2^ Linoleic acid ^3^ Alpha linolenic acid ^4^ Arachidonic acid ^5^ Eicosapentaenoic acid ^6^ Docosahexaenoic acid ^7^ Saturated fatty acid ^8^ Monounsaturated fatty acid ^9^ Polyunsaturated fatty acid.

**Table 4:** Primers used in qPCR studies.

Gene Name (Symbol) (GenBank Acc. No.) ^a,b,c^	Nucleotide sequence (5′–3′) ^d^	Amplicon Size (bp)	Efficiency (%) ^e^	
			Liver	Muscle
Acyl-coenzyme A oxidase 1, peroxisomal (*acox1*) (XM_021568072)	F: ACATACCACTGCCAGGTGTG	104	91.3	101.5
	R: GCGAGGAATTCGTACGTTCT			
Arachidonate 5-lipoxygenase a (*alox5a*) (XM_021580837)	F: GCTGGTGAAGATAGAGAAGCAG	110	81.0 ^e^	80.4 ^e^
	R: AGTGGAAGCAGGGGAACTCTA			
Carnitine O-palmitoyl transferase 1 alpha 1a (*cpt1a1a*) (AJ619768)	F: CATCCCAGCTGAGTGTCAGA	100	98.8	99.0
	R: GAAGCAATTGAAGGGGATGA			
Carnitine O-palmitoyl transferase 1 alpha 1b (*cpt1a1b*) (GU592679)	F: CCTACTTCAGAAGCGGCAAG	107	90.6	99.6
	R: CGGGTTGTCGATCTCGTATT			
Carnitine O-palmitoyl transferase 1 alpha 2a (*cpt1a2a*) (XM_021624450)	F: CCGCCCATAAAAGACACACT	123	ND	123.9 ^e^
	R: CAACCTGTTCCCCAGACTGT			
Carnitine O-palmitoyl transferase 1 alpha 2b (*cpt1a2b*) (XM_021565275)	F: ACCCCTGATGAGTTTGAACG	100	97.4	114.1 ^e^
	R: CCCAGAGAGCCTTGAGTTTG			
Carnitine O-palmitoyl transferase 1 beta 1a (*cpt1b1a*) (AJ606076)	F: TCGCTGTGATAGCCATCATG	99	109.2 ^e^	105.0
	R: TGTAGTCACTGACAGGCAGGG			
Carnitine O-palmitoyl transferase 1 beta 1b (*cpt1b1b*) (AF327058)	F: GAATGGTAAACTGGGGGTTAATG	108	92.6	94.8
	R: GTGTAGCCCAAAAGGAAGCA			
Cyclooxygenase-1a (*cox1a*) (AJ299018)	F: TGGGTCTGGGCATGTATCC	136	84.3	99.6
	R: CAATGCCAAACCTGACACAC			
Delta 5 fatty acyl desaturase (*fadsd5*) (XM_021598601)	F: AAATCCGGCTGGAACCACAA	114	92.0	105.2
	R: AAAAATGTTGGGCTTAGCGTG			
Delta 6 fatty acyl desaturase a (*fadsd6a*) (NM_001124287)	F: AGCCATCATTGATGTTGTCG	155	91.7	97.2
	R: CACAAACGTCTGGGGAAACT ^d^			
Delta 6 fatty acyl desaturase b (*fadsd6b*) (XM_021598609)	F: CTACTTATTCCAGTGTATTTCCAC	94	85.0	100.0
	R: GGTAGAAACTCATCGACCATGC			
ELOVL fatty acid elongase 2 (*elovl2*) (KM244737)	F: GATGCCTGCTCTTCCAGTTC	113	91.3	ND
	R: GCGACTGGACTTGATGGATT			
ELOVL fatty acid elongase 5a (*elovl5a*) (AY605100)	F: CTATGTCATCACACTTATTGCCC	123	92.0	91.5
	R: ACATGGCCATTCAATGAAGC			
ELOVL fatty acid elongase 5b (*elovl5b*) (XM_021576494)	F: ACAAGGCCAGCTGATTCAATT	123	89.1	ND
	R: GCAATAAGCGAGGCCATATAG			
60S ribosomal protein L32 (*rpl32*) (CF752566) ^a^	F: AGACCAAGCACATGCTACCC	149	83.1	89.0
	R: CCTCTCCACAATCAGCTTCC			
ATP-binding cassette sub-family F member 2 (*abcf2*) (CA383423) ^a^	F: CGTGTGGTGGATGACAAGAC	150	101.6	98.2
	R: GTCCAGGTCAATGCCAAACT			
Beta-actin (*actb*) (AF157514) ^a,b,c^	F: AGAGCTACGAGCTGCCTGAC	104	87.1	96.0
	R: GCAAGACTCCATACCGAGGA			
Elongation factor 1-alpha, oocyte form (*ef1a*) (CF752140) ^a,b^	F: CTTTGTGCCCATCTCTGGAT	122	82.4	94.0
	R: CCAGCAGAGTCACACCATTG			
Eukaryotic translation initiation factor 3 subunit D (*etif3d*) (CU070663) ^a^	F: CACCGAGCTGAAGAACAACA	108	103.5	98.1
	R: TTCGCGTGATAACGAGACAC			
Polyadenylate-binding protein cytoplasmic 1 (*pabpc1*) (CA355003) ^a,c^	F: AACCGCGCTGCCTACTACT	102	94.3	88.6
	R: GGGCATGTTCTGGAAGTGTT			

^a^ Candidate normalizers. ^b^ Expression levels of the transcripts of interest (TOIs) in the liver study were normalized to transcript expression levels of these two genes. ^c^ Expression levels of the TOIs in the muscle study were normalized to transcript expression levels of these two genes. ^d^ Primers are located in the coding sequence (CDS) with the exception of the reverse primer for *fadsd6a* which is located in the 3′untranslated region (UTR). ^e^ Amplification efficiencies were calculated using a 5-point 1:3 dilution series starting with cDNA representing 10 ng of input total RNA with the exception of *alox5a* (liver and muscle), *cpt1a2a* and *cpt1a2b* (muscle), and *cpt1b1a* (liver) which were calculated using a 4-point dilution series due to low expression levels. In the case of *alox5a* (80%) and *cpt1a2a* (124%), this low expression influenced the amplification efficiencies; however, as spacing was appropriate over this dilution series, and the variation in expression levels of these transcripts between the samples was not large, these assays were deemed acceptable. ND indicates that this transcript was not expressed at levels that could be accurately detected in this tissue. See Materials and Methods for details.

**Table 5:** Growth performance and whole-body analysis of rainbow trout fed experimental diets for 8 weeks ^1^.

Growth Parameters	FO	MO/CO	MO	*p*-Value
Initial weight ^2^	19.2 ± 2.9	18.7 ± 3.1	18.7 ± 2.8	0.419
Final weight ^3^	82.1 ± 14.4	75.6 ± 17.3	80.3 ± 15.8	0.064
Weight gain ^4^	62.7 ± 4.6	56.9 ± 2.0	61.6 ± 2.2	0.135
Initial length ^2^	11.7 ± 0.7	11.8 ± 0.8	11.7 ± 0.6	0.356
Final length ^3^	18.5 ± 1.0 ^a^	17.9 ± 1.2 ^b^	18.3 ± 1.2 ^a,b^	0.009
Initial CF ^5^	1.18 ± 0.1 ^a^	1.14 ± 0.1 ^b^	1.17 ± 0.1 ^a,b^	0.011
Final CF ^5^	1.3 ± 0.1	1.3 ± 0.1	1.3 ± 0.1	0.623
Initial VSI ^6^	12.3 ± 1.6	13.0 ± 1.1	12.9 ± 1.7	0.552
Final VSI ^6^	11.6 ± 1.3	11.2 ± 1.1	10.7 ± 1.4	0.169
SGR ^7^	2.6 ± 0.4	2.9 ± 0.3	2.5 ± 0.2	0.225
AFI ^8^	48.1 ± 2.5	43.5 ± 3.5	50.1 ± 3.6	0.107
FCR ^9^	0.8 ± 0.0	0.8 ± 0.0	0.8 ± 0.0	0.467
**Whole Body Analysis**				
Crude protein (%)	52.5 ± 2.1	51.7 ± 2.2	51.9 ± 2.0	0.897
Total fat (%)	32.5 ± 2.6	33.4 ± 3.2	31.9 ± 1.7	0.773
Ash (%)	7.2 ± 0.7	6.9 ± 0.4	6.9 ± 0.5	0.700
Calcium (%)	1.43 ± 0.18	1.37 ± 0.11	1.43 ± 0.16	0.848
Potassium (%)	1.10 ± 0.04	1.07 ± 0.05	1.05 ± 0.05	0.483
Magnesium (%)	0.09 ± 0.01	0.09 ± 0.01	0.09 ± 0.01	0.688
Phosphorus (%)	1.38 ± 0.11	1.30 ± 0.08	1.34 ± 0.07	0.598
Sodium (%)	0.27 ± 0.01	0.25 ± 0.03	0.22 ± 0.05	0.351
Zinc (ppm)	67.3 ± 7.1	61.8 ± 3.2	62.5 ± 5.3	0.462

^1^ Means with different superscripts indicate significant differences among treatments (*p* < 0.05) ^2^ Initial measurements are mean ± standard deviation, body weight (g fish^−1^), fork length (cm fish^−1^) *n*= 5 ^3^ Final measurements are mean ± standard deviation, body weight (g fish^−1^), fork length (cm fish^−1^) *n* = 5 ^4^ Weight gain (g fish^−1^) = final weight—initial weight (calculated by tank means). ^5^ Condition factor (CF) = body mass/length^3^ (calculated by individual fish). ^6^ Visceral somatic index (VSI, %) = 100 × (viscera mass/body mass). ^7^ Specific Growth Rate (SGR) = (ln (final body weight) − ln ((initial body weight))/56 days × 100. ^8^ apparent feed intake (AFI, g/fish) = (feed consumed, g)/(number of fish per tank) (calculated by tank means). ^9^ Feed conversion ratio (FCR) = (feed intake, g fish^−1^)/(weight gain, g fish^−1^) (calculated by tank means).

**Table 6:** Fatty acid content (µg/mg dry weight) and total lipid of rainbow trout liver, from week 0 (initial) and week 8 ^1^.

Fatty Acid	Initial	FO	MO/CO	MO	F-Value	*p*-Value
14:0	2.6 ± 0.7	1.3 ± 0.2 ^b^	1.8 ± 0.3 ^a^	1.8 ± 0.5 ^a^	8.87	0.001
14:1n-5	0.0 ± 0.0	0.0 ± 0.0	0.0 ± 0.0	0.0 ± 0.0	0.38	0.683
15:0	0.3 ± 0.1	0.2 ± 0.0 ^b^	0.4 ± 0.1 ^a^	0.3 ± 0.1 ^a^	39.99	0.000
16:0	31.0 ± 5.6	20.3 ± 3.4 ^b^	26.0 ± 4.3 ^a^	21.9 ± 3.2 ^b^	9.94	0.000
16:1n-7c	4.4 ± 2.4	1.7 ± 0.4	1.6 ± 0.6	1.7 ± 0.5	0.26	0.774
16:1n-7t	0.1 ± 0.0	0.1 ± 0.0 ^a^	0.0 ± 0.0 ^b^	0.0 ± 0.0 ^b^	9.56	0.000
17:0	0.4 ± 0.1	0.2 ± 0.1	0.2 ± 0.0	0.2 ± 0.0	3.13	0.054
17:1n-7	0.3 ± 0.1	0.1 ± 0.0	0.1 ± 0.0	0.1 ± 0.0	2.24	0.119
18:0	10.0 ± 1.7	8.0 ± 1.3 ^a,b^	8.8 ± 1.3 ^a^	7.3 ± 1.9 ^b^	3.52	0.039
18:1n-9c	18.8 ± 9.0	10.7 ± 2.4 ^ab^	11.8 ± 1.9 ^a^	9.6 ± 1.7 ^b^	4.12	0.023
18:1n-9t	0.1 ± 0.0	0.1 ± 0.0 ^a^	0.1 ± 0.0 ^b^	0.1 ± 0.0 ^b^	17.71	0.000
18:1n-12	0.3 ± 0.1	3.0 ± 0.1 ^a^	0.3 ± 0.1 ^a^	0.2 ± 0.0 ^b^	10.48	0.000
18:1n-7c	4.0 ± 1.0	1.8 ± 0.4	1.9 ± 0.4	2.0 ± 0.3	1.14	0.329
19:0	0.2 ± 0.0	0.1 ± 0.0 ^a^	0.1 ± 0.0 ^b^	0.1 ± 0.0 ^b^	37.67	0.000
19:1n-12	0.0 ± 0.0	0.0 ± 0.0	0.0 ± 0.0	0.0 ± 0.0	0.02	0.982
18:2n-6 (LNA) ^2^	3.4± 1.0	3.4 ± 0.5 ^b^	4.5 ± 0.6^a^	3.0 ± 0.6 ^b^	27.33	0.000
20:0	0.2 ± 0.1	0.3 ± 0.0 ^b^	0.3 ± 0.0 ^a^	0.2 ± 0.1^c^	23.95	0.000
18:3n-6	0.1 ± 0.0	0.0 ± 0.0	0.0 ± 0.0	0.0 ± 0.0	1.45	0.245
20:1n-15	0.1 ± 0.0	0.0 ± 0.0 ^a^	0.0 ± 0.0 ^b^	0.0 ± 0.0 ^b^	78.17	0.000
20:1	0.8 ± 0.3	0.3 ± 0.0 ^a^	0.1 ± 0.0 ^b^	0.1 ± 0.0 ^b^	124.72	0.000
20:1n-9	2.2 ± 0.7	3.1 ± 0.6 ^b^	3.8 ± 0.7 ^a^	2.6 ± 0.9 ^b^	9.88	0.000
18:3n-3 (ALA) ^3^	0.5 ± 0.2	1.6 ± 0.3 ^b^	2.6 ± 0.6 ^a^	1.5 ± 0.3 ^b^	34.95	0.000
18:4n-3	0.2 ± 0.1	0.1 ± 0.0 ^a^	0.1 ± 0.0 ^a^	0.1 ± 0.0 ^b^	13.12	0.000
20:2n-6	1.0 ± 0.3	1.7 ± 0.3 ^b^	2.1 ± 0.4 ^a^	1.3 ± 0.4 ^c^	17.72	0.000
22:0	0.1 ± 0.1	0.1 ± 0.0 ^a^	0.0 ± 0.0 ^b^	0.0 ± 0.0 ^b^	9.90	0.000
20:3n-6	0.9 ± 0.2	0.8 ± 0.1 ^a^	0.8 ± 0.2 ^a^	0.5 ± 0.2 ^b^	14.69	0.000
22:1n-11	0.3 ± 0.2	0.2 ± 0.1	0.1 ± 0.0	0.1 ± 0.1	1.02	0.371
22:1n-9	0.1 ± 0.1	0.2 ± 0.1 ^a,b^	0.2 ± 0.0 ^a^	0.1 ± 0.0 ^b^	8.36	0.001
20:3n-3	0.1 ± 0.1	0.7 ± 0.1 ^b^	1.1 ± 0.3 ^a^	0.6 ± 0.2 ^b^	23.08	0.000
20:4n-6 (ARA) ^4^	6.2 ± 1.1	4.2 ± 0.7 ^b^	5.7 ± 0.9 ^a^	4.8 ± 0.9 ^b^	11.31	0.000
20:5n-3 (EPA) ^5^	9.3 ± 1.5	6.2 ± 0.9 ^a^	2.4 ± 0.5 ^b^	2.0 ± 0.3 ^b^	191.76	0.000
24:1n-9	1.7 ± 0.3	1.1 ± 0.3	1.1 ± 0.4	1.0 ± 0.3	0.41	0.665
22:4n-6	0.2 ± 0.1	0.1 ± 0.1	0.2 ± 0.2	0.3 ± 0.3	1.00	0.375
22:5n-3	2.2 ± 0.4	1.5 ± 0.2	0.5 ± 0.2	0.5 ± 0.1	203.28	0.000
22:6n-3 (DHA) ^6^	66.4 ± 9.1	40.7 ± 5.8 ^b^	53.6 ± 6.9 ^a^	44.7 ± 8.6 ^b^	12.71	0.000
ΣSFA ^7^	44.7 ± 7.5	30.5 ± 4.8 ^b^	37.6 ± 5.1 ^a^	31.8 ± 4.9 ^b^	8.92	0.001
ΣMUFA ^8^	33.3 ± 13.8	19.6 ± 3.9 ^ab^	21.2 ± 3.1 ^a^	17.8 ± 3.3 ^b^	3.67	0.034
ΣMUFA ≥ 18	28.5 ± 11.3	17.7 ± 3.5 ^ab^	19.5 ± 2.7 ^a^	15.9 ± 3.0 ^b^	4.83	0.013
ΣMUFA < 18	5.3 ± 1.3	4.8 ± 0.8 ^ab^	5.4 ± 1.0 ^a^	4.0 ± 1.1 ^b^	7.80	0.001
ΣPUFA ^9^	90.7 ± 12.2	61.2 ± 8.2 ^b^	73.6 ± 9.6 ^a^	59.2 ± 10.8 ^b^	9.85	0.000
ΣC18 PUFA	4.2 ± 1.3	5.1 ± 0.8 ^b^	7.2 ± 1.1 ^a^	4.6 ± 0.8 ^b^	33.90	0.000
ΣC20 PUFA	17.5 ± 2.6	13.7 ± 1.8	12.1 ± 2.0	9.1 ± 1.8	22.47	0.000
ΣC22 PUFA	68.9 ± 9.4	42.4 ± 6.0 ^b^	54.4 ± 7.2 ^a^	45.4 ± 8.9 ^b^	10.59	0.000
ΣEPA & DHA	75.8 ± 10.3	47.0 ± 6.6 ^b^	50.6 ± 7.3 ^a^	46.7 ± 8.8 ^b^	7.20	0.002
Σn-3	78.8 ± 10.6	50.9 ± 7.0 ^b^	60.3 ± 8.0 ^a^	49.2 ± 9.0 ^b^	8.17	0.001
Σn-6	11.9 ± 1.9	10.4 ± 1.4 ^b^	13.4 ± 1.9 ^a^	9.9 ± 2.0 ^b^	16.73	0.000
Σodd chain	1.2 ± 03	0.7 ± 0.1 ^b^	0.8 ± 0.1 ^a^	0.7 ± 0.1 ^ab^	6.95	0.003
n-3/n-6	6.7 ± 0.7	4.9 ± 0.3 ^a^	4.5 ± 0.4 ^b^	5.0 ± 0.4 ^a^	6.69	0.003
EPA/ARA	1.5 ± 0.3	1.5 ± 0.1^a^	0.4 ± 0.0 ^b^	0.4 ± 0.1 ^b^	860.23	0.000
**Total Lipid**									
Lipid % ww	4.7 ± 0.6	4.2 ± 1.0	3.9 ± 0.5	4.2 ± 1.0	0.59	0.560
Lipid % dw	20.1 ± 2.7	17.4 ± 4.0	16.2 ± 2.3	17.5 ± 3.7	0.62	0.543
Lipid (µg/mg) ww	39.7 ± 6.9	26.9 ± 3.7 ^b^	32.0 ± 3.7 ^a^	26.2 ± 4.7 ^b^	8.93	0.001
Lipid (µg/mg) dw	168.7 ± 29.8	111.2 ±15.1 ^b^	132.5 ± 16.9 ^a^	108.7 ± 18.5^b^	8.85	0.001

^1^ Data expressed as µg FAME/mg (dry weight), values are means (*n* = 3 per treatment) ± standard deviation. Means with different superscripts indicate significant differences based on Tukey’s post-hoc test following a one-way ANOVA. FO, fish oil; MO/CO, microbial oil/camelina oil blend; MO, microbial oil (*Schizochytrium* sp. T-18). ^2^ Linoleic acid ^3^ Alpha linolenic acid ^4^ Arachidonic acid ^5^ Eicosapentaenoic acid ^6^ Docosahexaenoic acid ^7^ Saturated fatty acid ^8^ Monounsaturated fatty acid ^9^ Polyunsaturated fatty acid.

**Table 7:** Fatty acid content (µg/mg dry weight) and total lipid of rainbow trout muscle, from week 0 (initial) and week 8 ^1^.

Fatty Acid	Initial	FO	MO/CO	MO	F-Value	*p*-Value
14:0	5.1 ± 1.5	7.1 ± 2.3 ^b^	8.9 ± 3.3 ^ab^	10.9 ± 2.5 ^a^	7.35	0.002
14:1n-5	0.1 ± 0.0	0.1 ± 0.1 ^b^	0.1 ± 0.0 ^ab^	0.1 ± 0.0 ^a^	3.83	0.030
15:0	0.4 ± 0.1	0.5 ± 0.2 ^b^	1.0 ± 0.4 ^a^	1.2 ± 0.3 ^a^	25.99	0.000
16:0	26.6 ± 7.3	39.6 ± 12.1 ^b^	47.7 ± 15.2 ^ab^	51.8 ± 10.7 ^a^	3.56	0.037
16:1n-7c	8.7 ± 2.7	11.3 ± 4.0	9.7 ± 3.9	10.8 ± 2.6	0.73	0.490
16:1n-7t	0.1 ± 0.0	0.2 ± 0.1 ^a^	0.1 ± 0.0 ^b^	0.0 ± 0.0 ^b^	33.25	0.000
17:0	0.3 ± 0.1	0.5 ± 0.2	0.4 ± 0.1	0.4 ± 0.1	1.05	0.361
17:1n-7	0.2 ± 0.1	0.3 ± 0.1	0.2 ± 0.1	0.2 ± 0.0	2.10	0.135
18:0	5.6 ± 1.6	8.4 ± 2.7	8.7 ± 2.7	8.3 ± 1.6	0.11	0.894
18:1n-9c	31.0 ± 9.7	40.1 ± 15.0	44.5 ±17.4	39.1 ± 9.6	0.59	0.557
18:1n-9t	0.2 ± 0.1	0.2 ± 0.1 ^a^	0.1 ± 0.0 ^b^	0.1 ± 0.0 ^b^	9.90	0.000
18:1n-12	0.9 ± 0.3	0.7 ± 0.2	0.8 ± 0.3	0.7 ± 0.1	0.38	0.684
18:1n-7c	4.7 ± 1.4	5.1 ± 1.8	5.8 ± 2.2	6.0 ± 1.3	1.08	0.350
18:1n-7t	0.3 ± 0.2	0.6 ± 0.3 ^a^	0.1 ± 0.1 ^b^	0.0 ± 0.0 ^b^	71.09	0.000
19:1n-12	0.3 ± 0.1	0.7 ± 0.3 ^a^	0.1 ± 0.0 ^b^	0.0 ± 0.0 ^b^	83.41	0.000
18:2n-6 (LNA) ^2^	11.6 ± 3.4	18.9 ± 6.5 ^b^	26.4 ± 10.3 ^a^	21.7 ± 5.3 ^ab^	3.59	0.036
20:0	0.2 ± 0.1	0.5 ± 0.2 ^b^	0.7 ± 0.3 ^a^	0.5 ± 0.1 ^b^	4.72	0.014
18:3n-6	0.3 ± 0.1	0.3 ± 0.1	0.3 ± 0.1	0.3 ± 0.1	0.57	0.571
20:1n-15	0.0 ± 0.0	0.0 ± 0.0 ^a^	0.0 ± 0.0 ^b^	0.0 ± 0.0 ^b^	23.89	0.000
20:1	2.6 ± 0.8	1.2 ± 0.5 ^a^	1.1 ± 0.4 ^ab^	0.8 ± 0.2 ^b^	3.69	0.033
20:1n-9	2.2 ± 0.7	7.7 ± 2.7 ^b^	11.7 ± 4.4 ^a^	8.8 ± 2.2 ^b^	6.01	0.005
18:3n-3 (ALA) ^3^	2.4 ± 0.7	13.8 ± 4.5 ^b^	23.1 ± 8.7 ^a^	16.1 ± 4.0 ^b^	9.31	0.000
18:4n-3	1.4 ± 0.4	2.2 ± 0.7 ^a^	1.4 ± 0.5 ^b^	1.1 ± 0.3 ^b^	19.14	0.000
20:2n-6	0.9 ± 0.3	2.2 ± 0.6 ^ab^	2.7 ± 1.0 ^a^	2.0 ± 0.4 ^b^	4.35	0.019
22:3n-3	0.0 ± 0.0	0.1 ± 0.1 ^b^	0.2 ± 0.1 ^a^	0.2 ± 0.1 ^ab^	7.45	0.002
22:0	0.1 ± 0.0	0.2 ± 0.1	0.2 ± 0.1	0.2 ± 0.0	1.13	0.332
20:3n-6	0.5 ± 0.2	0.6 ± 0.2	0.7 ± 0.2	0.6 ± 0.1	1.09	0.347
22:1n-11	2.3 ± 0.7	2.4 ± 0.9	2.8 ± 1.1	2.6 ± 0.6	0.88	0.422
22:1n-9	0.4 ± 0.1	1.2 ± 0.4 ^b^	1.8 ± 0.7 ^a^	1.4 ± 0.3 ^ab^	5.84	0.006
20:3n-3	0.2 ± 0.1	1.1 ± 0.3 ^b^	1.9 ± 0.7 ^a^	1.3 ± 0.3 ^a^	11.82	0.000
20:4n-6 (ARA) ^4^	1.3 ± 0.3	1.4 ± 0.3	1.4 ± 0.3	1.5 ± 0.2	0.64	0.534
22:2n-6	0.1 ± 0.0	0.2 ± 0.1 ^a^	0.2 ± 0.1 ^ab^	0.1 ± 0.0 ^b^	4.34	0.019
24:0	0.1 ± 0.0	0.1 ± 0.1	0.2 ± 0.1	0.1 ± 0.1	1.61	0.213
20:5n-3 (EPA) ^5^	7.4 ± 1.8	9.5 ± 2.2 ^a^	3.4 ± 0.8 ^b^	3.0 ± 0.5 ^b^	105.04	0.000
24:1n-9	0.5 ± 0.1	0.8 ± 0.3	1.0 ± 0.3	0.9 ± 0.2	1.59	0.215
22:4n-6	0.2 ± 0.0	0.2 ± 0.1	0.2 ± 0.1	0.2 ± 0.1	0.78	0.467
22:5n-3	2.0 ± 0.5	2.6 ± 0.8 ^a^	1.0 ± 0.3 ^b^	0.9 ± 0.2 ^b^	60.54	0.000
22:6n-3 (DHA) ^6^	20.6 ± 4.6	23.3 ± 3.5 ^c^	38.7 ± 8.8 ^b^	47.1 ± 7.7 ^a^	43.89	0.000
ΣSFA ^7^	38.5 ± 10.6	56.9 ± 17.7 ^a^	67.8 ± 22.1 ^ab^	73.5 ± 15.3 ^b^	3.10	0.056
ΣMUFA ^8^	54.6 ± 16.6	72.7 ± 26.5	80.1 ± 30.8	72.0 ± 17.1	0.46	0.632
ΣMUFA ≥ C18	45.4 ± 13.8	61.0 ± 22.3	69.9 ± 26.9	60.7 ± 14.4	0.87	0.426
ΣMUFA < C18	8.3 ± 2.5	14.2 ± 5.1	18.7 ± 7.1	14.7 ± 3.5	3.06	0.057
ΣPUFA ^9^	48.9 ± 12.0	76.5 ± 19.3 ^b^	101.3 ± 31.6 ^a^	95.9 ± 18.8 ^ab^	4.44	0.018
ΣC18 PUFA	15.7 ± 4.6	35.3 ± 11.7 ^b^	51.2 ± 19.6 ^a^	39.2 ± 9.6 ^ab^	5.00	0.011
ΣC20 PUFA	10.3 ± 2.6	14.8 ± 3.4 ^a^	10.0 ± 2.9 ^b^	8.3 ± 1.4 ^b^	22.94	0.000
ΣC22 PUFA	22.9 ± 5.1	26.3 ± 4.3 ^c^	40.1 ± 9.2 ^b^	48.3 ± 7.9 ^a^	33.51	0.000
ΣEPA & DHA	28.0 ± 6.3	32.8 ± 5.5 ^c^	42.1 ± 9.5 ^b^	50.1 ± 8.1 ^a^	17.97	0.000
Σn-3	34.0 ± 7.9	52.6 ± 11.6 ^b^	69.5 ± 19.6 ^a^	69.5 ± 12.7 ^a^	6.30	0.004
Σn-6	14.9 ± 4.2	23.9 ± 7.7	31.8 ± 12.0	26.4 ± 6.2	3.05	0.058
ΣOdd chain	1.3 ± 0.4	2.1 ± 0.7	1.8 ± 0.7	2.0 ± 0.4	0.92	0.408
n-3/n-6	2.3 ± 0.3	2.3 ± 0.3 ^b^	2.3 ± 0.2 ^b^	2.7 ± 0.2 ^a^	13.36	0.000
EPA/ARA	5.9 ± 0.4	6.9 ± 0.5 ^a^	2.1 ± 0.2 ^c^	2.4 ± 0.2 ^b^	1039.80	0.000
EPA + DHA/100g	607.0 ± 141	799.4 ± 151.6 ^c^	1007.8 ± 241.2 ^b^	1252.3 ± 231.0 ^a^	17.9	0.000
**Total lipid**						
Lipid % ww	3.6 ± 0.7	4.6 ± 1.2	4.8 ± 1.1	5.3 ± 1.2	1.34	0.272
Lipid % dw	16.6 ± 3.2	19.0 ± 4.5	20.2 ± 4.3	21.2 ± 4.4	0.92	0.406
Lipid (µg/mg) ww	30.8 ± 8.8	50.4 ± 16.6	59.8 ± 20.9	60.4 ± 14.3	1.55	0.223
Lipid (µg/mg) dw	141.9 ± 38.8	206.1 ± 63.2	249.2 ± 84.1	241.3 ± 50.8	1.73	0.189

^1^ Data expressed as µg FAME/mg (dry weight), values are means (*n* = 3 per treatment) ± standard deviation. Means with different superscripts indicate significant differences based on Tukey’s post-hoc test following a one-way ANOVA. FO, fish oil; MO/CO, microbial oil/camelina oil blend; MO, microbial oil (*Schizochytrium* sp. T-18). ^2^ Linoleic acid ^3^ Alpha linolenic acid ^4^ Arachidonic acid ^5^ Eicosapentaenoic acid ^6^ Docosahexaenoic acid ^7^ Saturated fatty acid ^8^ Monounsaturated fatty acid ^9^ Polyunsaturated fatty acid.

## Data Availability

The data presented in this study are available in the Supplementary Materials.

## Supplementary Materials

The following are available online at https://www.mdpi.com/article/10.3390/ani11041185/s1, Figure S1: Principal coordinate ordination plot relating individual fatty acid profiles with relative expression of targeted transcripts from liver of rainbow trout fed either the fish oil (FO) control diet, microbial oil/camelina oil (MO/CO) diet, or the microbial oil (MO) diet, Figure S2: Principal coordinate ordination plot relating individual fatty acid profiles with relative expression of targeted transcripts from muscle of rainbow trout fed either the fish oil (FO) control diet, microbial oil/camelina oil (MO/CO) diet, or the microbial oil (MO) diet, Table S1: Correlation analyses (Pearson correlation coefficient, R) comparing relationships among transcript expression (RQ values) of targeted transcripts from the study with relevant n-3 and n-6 PUFA in liver and muscle.
