# Supplementary material for: Schizochytrium sp. (T18) Oil as a Fish Oil Replacement in Diets for Juvenile Rainbow Trout (Oncorhynchus mykiss): Effects on Growth Performance, Tissue Fatty Acid Content, and Lipid-Related Transcript Expression
Source: Animals (Basel). 2021 Apr 20;11(4):1185. doi: 10.3390/ani11041185 (PMC8074903; doi:10.3390/ani11041185)
Supplement: Supplementary file 1 [file animals-11-01185-s001.zip › animals-1160805-supplementary.pdf]

Supplementary Information

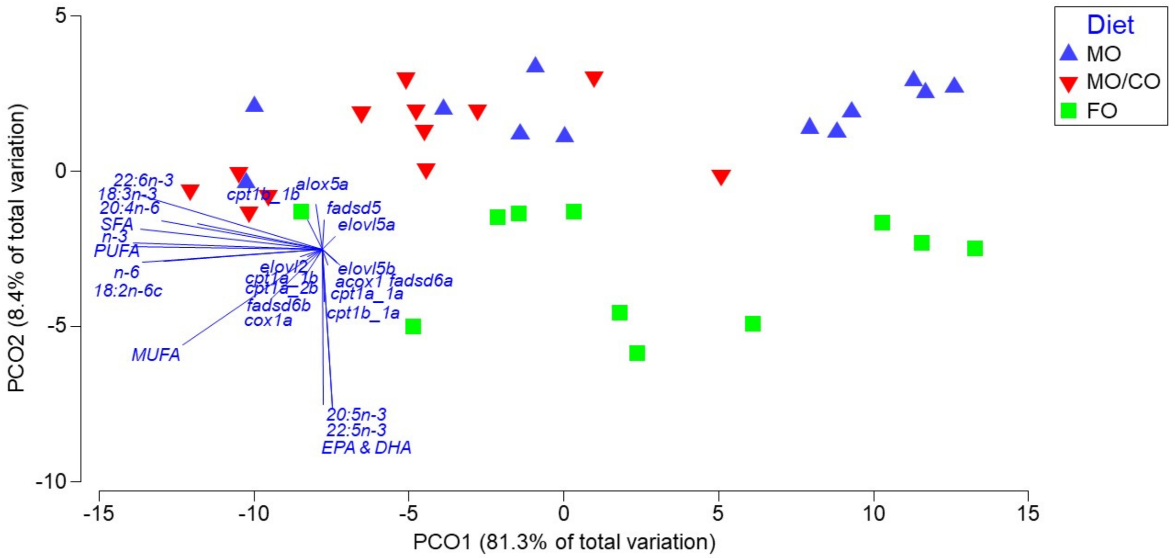

Supplementary Figure S1. Principal coordinate ordination plot relating individual fatty acid profiles with relative expression of targeted transcripts from liver of rainbow trout fed either the fish oil (FO) control diet, microbial oil/camelina oil (MO/CO) diet, or the microbial oil (MO) diet.

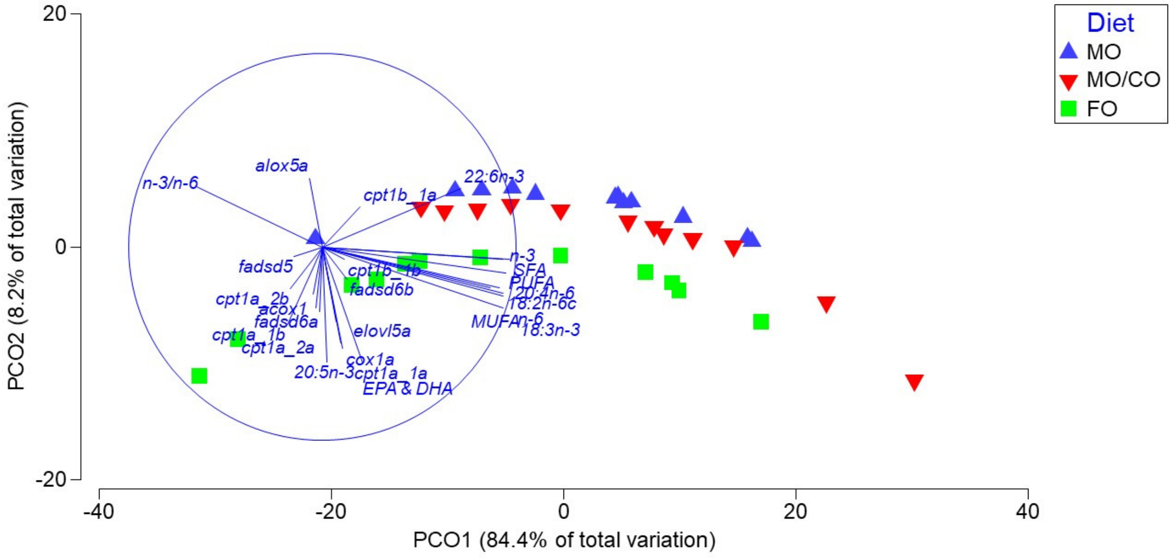

Supplementary Figure S2. Principal coordinate ordination plot relating individual fatty acid profiles with relative expression of targeted transcripts from muscle of rainbow trout fed either the fish oil (FO) control diet, microbial oil/camelina oil (MO/CO) diet, or the microbial oil (MO) diet.

Supplementary Table S1. Correlation analyses (Pearson correlation coefficient, R) comparing relationships among transcript expression (RQ values) of targeted transcripts from the study with relevant n-3 and n-6 PUFA in liver and muscle.

		Liver		Muscle	
**Transcript**	**Fatty Acid**	**Pearson R**	***p*-Value**	**Pearson R**	***p*-Value**
elovl2	DHA	0.0573	0.744		
	EPA	-0.0992	0.571		
	ARA	-0.0095	0.957		
	ALA	0.0215	0.903		
	LNA	0.157	0.369		
	n-3	0.0235	0.894		
	n-6	0.0587	0.737		
	18:4n-3	0.0552	0.753		
	20:3n-3	-0.108	0.535		
	22:5n-3	-0.174	0.316		
	18:3n-6	0.250	0.147		
	20:3n-6	0.156	0.372		
elovl5a	DHA	0.000979	0.996	0.00345	0.984
	EPA	-0.150	0.389	0.103	0.552
	ARA	0.0264	0.881	0.136	0.429
	ALA	-0.302	0.0781	0.287	0.0901
	LNA	-0.181	0.297	0.237	0.165
	n-3	-0.0747	0.670	0.168	0.327
	n-6	-0.113	0.517	0.235	0.168
	18:4n-3	-0.108	0.538	0.0933	0.588
	20:3n-3	-0.0799	0.648	0.308	0.0673
	22:5n-3	-0.156	0.372	0.104	0.548
	18:3n-6	0.140	0.424	-0.0100	0.954
	20:3n-6	-0.000124	0.999	0.177	0.301
elovl5b	DHA	-0.0707	0.686		
	EPA	-0.043	0.802		
	ARA	0.0136	0.938		
	ALA	-0.174	0.316		
	LNA	-0.0594	0.735		
	n-3	-0.100	0.566		
	n-6	-0.0652	0.710		
	18:4n-3	-0.0651	0.710		
	20:3n-3	-0.0837	0.633		
	22:5n-3	-0.0496	0.777		
	18:3n-6	0.216	0.212		
	20:3n-6	0.101	0.563		
fadsd5	DHA	0.0448	0.798	-0.101	0.559
	EPA	-0.256	0.138	0.0763	0.658
	ARA	0.0882	0.614	-0.0265	0.878
	ALA	-0.161	0.355	-0.106	0.540
	LNA	-0.0531	0.762	-0.0962	0.577
	n-3	-0.0520	0.767	-0.106	0.537
	n-6	-0.0350	0.842	-0.107	0.535
	18:4n-3	-0.0893	0.610	-0.0456	0.792
	20:3n-3	-0.024	0.889	-0.123	0.476
	22:5n-3	-0.313	0.0672	0.0200	0.908
	18:3n-6	0.0810	0.644	-0.170	0.323
	20:3n-6	-0.132	0.449	-0.186	0.278
fadsd6a	DHA	-0.106	0.544	-0.171	0.320
	EPA	-0.0437	0.803	0.279	0.0995

	ARA	-0.0370	0.833	0.0566	0.743
	ALA	-0.167	0.337	-0.0151	0.930
	LNA	-0.0481	0.784	0.00519	0.976
	n-3	-0.134	0.442	-0.0483	0.780
	n-6	-0.0529	0.763	0.0191	0.912
	18:4n-3	-0.0393	0.822	0.277	0.102
	20:3n-3	-0.0442	0.801	-0.0280	0.871
	22:5n-3	-0.107	0.541	0.239	0.160
	18:3n-6	0.109	0.533	0.192	0.261
	20:3n-6	0.0896	0.609	0.110	0.523
fadsd6b	DHA	0.0571	0.745	0.00447	0.979
	EPA	-0.0317	0.857	0.0368	0.831
	ARA	0.0383	0.827	0.0822	0.634
	ALA	0.117	0.503	0.350	**0.0362**
	LNA	0.183	0.293	0.276	0.103
	n-3	0.0543	0.757	0.181	0.292
	n-6	0.132	0.448	0.267	0.116
	18:4n-3	0.197	0.257	0.0778	0.652
	20:3n-3	0.0161	0.927	0.398	**0.0162**
	22:5n-3	-0.0978	0.576	-0.0135	0.938
	18:3n-6	0.214	0.216	-0.0289	0.867
	20:3n-6	0.224	0.196	0.0977	0.571
acox1	DHA	-0.0412	0.814	-0.111	0.518
	EPA	0.158	0.364	0.191	0.265
	ARA	-0.0963	0.582	0.0274	0.874
cpt1a1a	DHA	-0.0515	0.769	-0.106	0.537
	EPA	0.0886	0.613	0.191	0.264
	ARA	-0.0249	0.887	0.135	0.432
cpt1a1b	DHA	0.0966	0.581	-0.455	**0.00531**
	EPA	0.103	0.555	0.405	**0.0143**
	ARA	0.0185	0.916	-0.118	0.495
cpt1a2a	DHA			-0.243	0.153
	EPA			0.219	0.199
	ARA			-0.0954	0.580
cpt1a2b	DHA	0.0482	0.783	-0.370	0.0263
	EPA	0.211	0.224	0.247	0.146
	ARA	0.0178	0.919	-0.184	0.284
cpt1b1a	DHA	-0.138	0.430	0.418	**0.0113**
	EPA	0.420	**0.0120**	-0.458	**0.00494**
	ARA	-0.188	0.280	0.142	0.409
cpt1b1b	DHA	0.125	0.473	0.0363	0.833
	EPA	0.127	0.467	-0.0282	0.870
	ARA	0.0293	0.867	0.0499	0.772
alox5a	DHA	0.173	0.321	0.210	0.220
	EPA	-0.170	0.328	-0.191	0.264
	ARA	0.0334	0.849	-0.0420	0.808
cox1a	DHA	0.174	0.318	-0.164	0.340
	EPA	0.130	0.455	0.251	0.139
	ARA	0.145	0.406	0.123	0.476
	16:0	0.0439	0.802	0.0655	0.704
	18:0	0.317	0.0638	0.235	0.169

Supplementary Table S2. Correlation analyses (Pearson correlation coefficient, R) comparing relationships among Table 3. and n-6 PUFA in the diet.

		Liver		Muscle	
**Transcript**	**Diet FA**	**Pearson R**	***p*-Value**	**Pearson R**	***p*-Value**
elovl2	DHA	-0.069	0.696		
	EPA	-0.014	0.937		
	ARA	-0.030	0.864		
	ALA	0.128	0.463		
	LNA	0.097	0.577		
elovl5a	DHA	0.306	0.074	-0.210	0.218
	EPA	-0.171	0.326	0.033	0.848
	ARA	-0.136	0.435	-0.004	0.981
	ALA	-0.161	0.355	0.262	0.122
	LNA	-0.043	0.805	0.172	0.316
elovl5b	DHA	0.120	0.493		
	EPA	-0.078	0.654		
	ARA	-0.067	0.704		
	ALA	-0.043	0.806		
	LNA	0.002	0.990		
fadsd5	DHA	0.233	0.179	-0.055	0.751
	EPA	-0.191	0.271	0.125	0.469
	ARA	-0.175	0.315	0.134	0.437
	ALA	-0.015	0.932	-0.140	0.416
	LNA	0.070	0.690	-0.152	0.375
fadsd6a	DHA	-0.054	0.760	-0.288	0.088
	EPA	0.058	0.740	0.288	0.089
	ARA	0.057	0.746	0.276	0.104
	ALA	-0.022	0.902	-0.075	0.665
	LNA	-0.040	0.820	-0.175	0.307
fadsd6b	DHA	-0.245	0.156	-0.210	0.240
	EPA	0.035	0.840	-0.047	0.783
	ARA	-0.009	0.960	-0.096	0.578
	ALA	0.309	0.071	0.393	**0.018**
	LNA	0.206	0.235	0.299	0.077
acox1	DHA	-0.179	0.304	-0.171	0.320
	EPA	0.218	0.209	0.234	0.170
	ARA	0.216	0.212	0.237	0.164
cpt1a1a	DHA	0.030	0.865	-0.413	**0.012**
	EPA	0.076	0.663	0.184	0.284
	ARA	0.095	0.588	0.130	0.451
cpt1a1b	DHA	-0.319	0.062	-0.558	**<0.001**
	EPA	0.271	0.116	0.468	**0.004**
	ARA	0.249	0.148	0.431	**0.009**
cpt1a2a	DHA			-0.432	**0.009**
	EPA			0.268	0.114
	ARA			0.224	0.189
cpt1a2b	DHA	-0.161	0.355	-0.408	**0.014**
	EPA	0.270	0.117	0.304	0.071
	ARA	0.281	0.102	0.271	0.110
cpt1b1a	DHA	-0.632	**<0.001**	0.409	**0.013**
	EPA	0.530	**0.001**	-0.411	**0.013**
	ARA	0.486	**0.003**	-0.395	**0.017**
cpt1b1b	DHA	-0.039	0.825	-0.087	0.615
	EPA	0.208	0.230	0.026	0.878

	ARA	0.234	0.176	0.013	0.939
alox5a	DHA	0.284	0.099	0.407	**0.014**
	EPA	-0.074	0.672	-0.216	0.207
	ARA	-0.028	0.872	-0.168	0.327
cox1a	DHA	-0.484	**0.003**	-0.440	**0.007**
	EPA	0.183	0.292	0.239	0.160
	ARA	0.114	0.514	0.189	0.271
